# Methodologies used in the development of the international classification of functioning, disability and health core sets: a systematic scoping review

**DOI:** 10.1136/bmjopen-2026-123955

**Published:** 2026-10-05

**Authors:** Lorenzo Billiet, Stijn De Baets, Dominique Van de Velde, Ruth M A van Nispen, Hilde P A van der Aa

**Affiliations:** 1 Amsterdam UMC Locatie AMC, Amsterdam, The Netherlands; 2 Amsterdam Public Health Research Institute, Amsterdam, The Netherlands; 3 Faculty of Medicine and Health Sciences, Ghent University, Ghent, Belgium; 4 Department of Rehabilitation Sciences, Ghent University, Gavere - asper, Belgium; 5 Ghent University Department of Rehabilitation Sciences, Ghent, Flanders, Belgium; 6 Ophthalmology, Amsterdam UMC Locatie VUmc, Amsterdam, The Netherlands

**Keywords:** OPHTHALMOLOGY, Review, Health Services, Methods, Occupational Health

## Abstract

**Abstract:**

**Objectives:**

To systematically examine the methodologies used in the development of International Classification of Functioning, Disability and Health (ICF) Core Sets (CSs) across all developmental phases, and to identify methodological variation in stakeholder involvement, geographical representation, consensus procedures and validation approaches to inform the future development of ICF CSs.

**Design:**

Scoping review conducted in accordance with the Preferred Reporting Items for Systematic Reviews and Meta-Analyses Extension for Scoping Reviews (PRISMA-ScR) and Joanna Briggs Institute methodological guidance.

**Setting:**

Literature published between January 2001 and July 2024. Publications were identified through PubMed, supplemented by reference list screening and author contact where necessary.

**Participants:**

We identified 170 relevant publications related to the development, validation and implementation, covering 34 CS across diverse health conditions. Eligible publications comprised preparatory studies (eg, systematic reviews, patient perspective studies, expert surveys), protocol/discussion papers, consensus conference reports, validation and implementation studies.

**Results:**

Most publications originated from Europe. The comprehensive CS averaged 97 ICF categories (SD 29) and the brief CS 26 (SD 12). In 38% of the studies, the focus was only on validity research. Variations were observed in patient and expert involvement during development. Considerable variation was observed in stakeholder involvement, geographical representation, consensus procedures and validation approaches across developmental phases.

**Conclusion:**

This review demonstrates substantial methodological diversity in ICF CS development across the different phases. While the developmental framework proposed by Selb *et al* was generally reflected across studies, considerable variation existed in how developmental phases were operationalised. Future ICF CS developments may benefit from stakeholder involvement, more balanced geographical representation, and continued attention to validation and implementation activities.

**STRENGTHS AND LIMITATIONS OF THIS STUDY**

- This systematic scoping review provides a comprehensive overview of International Classification of Functioning, Disability and Health (ICF) Core Set development by including all phases of the process (preparatory, consensus and validation), rather than focusing on a single stage.
- A structured and transparent methodology was applied in accordance with Preferred Reporting Items for Systematic Reviews and Meta-Analyses Extension for Scoping Reviews and established scoping review guidelines, enhancing reproducibility.
- The use of predefined ICF Core Set development phases as an analytical framework allowed for consistent comparison across studies.
- The literature search relied primarily on PubMed, which may have resulted in missing relevant methodological publications indexed in other databases.
- However, as ICF Core Sets are centrally documented and publicly accessible via the official ICF Core Sets platform, the likelihood of missing entire Core Sets was considered low and was mitigated through reference screening and author contact.

## Introduction

 The International Classification of Functioning, Disability and Health (ICF), that was endorsed by the WHO in 2001, provides a comprehensive framework to describe human functioning.^1^ The ICF can be used in clinical practice, policy making and the field of research.^2^

However, due to the extensive nature of the ICF, which consists of 1424 categories, to enhance feasibility and clinical usability, there is a need to shortlist these categories for specific health conditions to describe the functioning and disabilities of particular groups of people condition-specific ICF Core Sets (CSs) were developed.^3^ These CSs aim to identify the most relevant ICF categories needed to describe functioning, disability and contextual factors for specific health conditions while retaining the biopsychosocial perspective of the ICF framework. There are comprehensive CS, consisting of all ICF categories relevant for a specific condition, and brief CS, which are more compressed versions of the comprehensive CS.^3^

The development of an ICF CS is commonly guided by the framework described by Selb *et al*,^3^ which provides methodological recommendations for the creation of condition-specific CSs. This framework consists of three interrelated phases. First, a preparatory phase gathers evidence from different perspectives, including the research, patient and expert perspectives, through systematic reviews, qualitative studies and expert surveys. Second, an international consensus process is used to determine which ICF categories should be included in the comprehensive and brief CSs. Third, the resulting CSs undergo validation and implementation studies to evaluate their relevance, applicability and feasibility across settings and populations. Because this framework has become the dominant approach for ICF Core Set development, it was used as the organising structure for the present review (figure 1).

**Figure 1:**
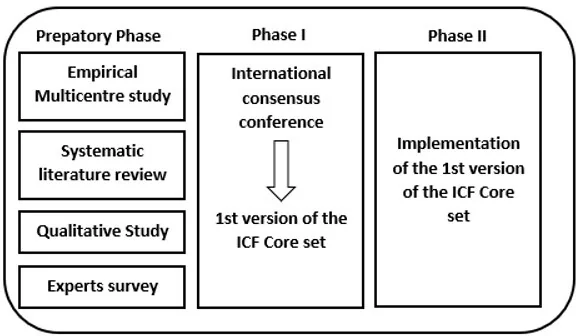
Phases in the development of an ICF CS.^3 12^ ICF CS, International Classification of Functioning, Disability and Health Core Set.

A key methodological component of many ICF CS development studies is the use of the ICF linking rules developed by Cieza *et al*.^4 5^ These rules provide a standardised procedure for linking concepts identified in research evidence, patient interviews, questionnaires and expert surveys to the most appropriate ICF categories. The linking rules support consistency and transparency across preparatory studies and have become a cornerstone of ICF CS methodology.

Previous literature reviews have been performed on the development of ICF CS. Yen *et al*^6^ carried out a systematic review of the development of ICF CS from 2001 to 2012 to provide more background information on the development process of ICF CS. More recently, Karlosson & Gustalsson^7^ published a scoping review on the validation of ICF CS developed between 2001 and 2019. They only focused on phase II in the development of ICF CS. They showed that only 66% of the existing ICF CS have been validated from the perspectives of different stakeholders and implemented in practice, mostly based on a European perspective. They concluded that there is a need for more validation research from both the patient and professional perspective in various global regions.

While general guidelines exist, they are not specific enough for researchers to appreciate the actual steps and methodology that need to be considered in developing a reliable and valid ICF CS. Consequently, it appears that different researchers have adopted diverse approaches in crafting an ICF CS.^8–10^ Given the substantial increase in the number of ICF CS developed over the past decade, a contemporary synthesis of development methodologies is needed. Such an overview can identify methodological trends, areas of inconsistency and potential gaps in current practice, thereby providing guidance for future CS development initiatives. Therefore, this systematic scoping review aimed to examine the methods and techniques used in the development of ICF CS between 2001 and 2024 across all developmental phases. By mapping methodological variation, stakeholder involvement, geographical representation and validation approaches, this review seeks to provide practical recommendations for the future development of robust, internationally applicable ICF CS.

## Materials & methods

To conduct the systematic scoping review, we followed the Preferred Reporting Items for Systematic Reviews and Meta-Analyses extension for scoping reviews (PRISMA-ScR)^11^ and Joanna Briggs Institute Reviewers guidelines^12^ and the methodological recommendations of Peters *et al*.^13^ The method of a systematic scoping review was used because it provided the opportunity to map the breadth and depth of knowledge in the development of any CS and provided opportunities to identify gaps in the available knowledge. This approach was considered particularly appropriate because the objective of the review was to examine and compare the wide range of methodologies used across the preparatory, consensus and validation phases of ICF CS development rather than to evaluate intervention effectiveness or synthesise outcome data.

### Search strategy

As ICF CSs are centrally documented and publicly accessible via the official ICF Research Branch website, we expected that the identification of existing CSs would be relatively complete when using PubMed combined with reference screening and author contact. However, the objective of this review extended beyond identifying CSs and included examining the methodological studies underpinning their development. To improve methodological coverage, reference lists of included studies were screened and additional publications were sought through direct contact with authors when necessary. Nevertheless, it remains possible that methodological publications not indexed in PubMed or not linked through reference chains were missed.

Relevant publications were identified in PubMed using standardised search terms (MeSH) combined with Boolean expressions (online supplemental appendix 2). Additional publications were identified by checking the reference lists of the relevant publications. The search was conducted in July 2024 and was limited to the years between January 2001 and July 2024. Relevant studies were manually selected by reviewing titles and abstracts. After selection, the full publications were read and relevant data were extracted from the included studies.

### Inclusion and exclusion criteria

Because the ICF was officially launched in 2001, only publications reporting on any phase of the development of ICF CS that were published between January 2001 and July 2024 were included. Eligible publications included preparatory studies (eg, systematic reviews, patient perspective studies and expert surveys), protocol or invitation papers, consensus conference reports, validation studies and implementation studies. Additionally, publications had to be written in English and the research had to be performed on human subjects. Publications were excluded if they were not related to the development of CS.

### Selection process

The literature search generated a total of 460 publications in PubMed. Additionally, 47 publications were found via reference lists. Figure 2 shows the PRISMA flow diagram of the selection process for this review.

**Figure 2:**
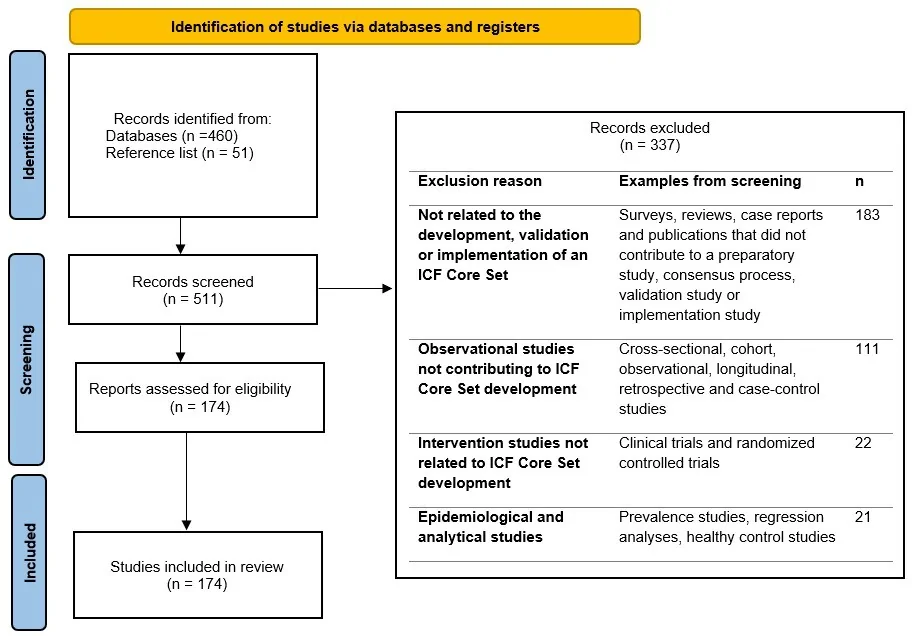
Flow diagram of the literature review.^33^ ICF, International Classification of Functioning, Disability and Health.

The titles and abstracts of all publications were systematically screened to assess their eligibility based on the inclusion criteria. To ensure the accuracy of the exclusion process, 10% of the excluded articles were randomly re-evaluated independently by a senior researcher (SDB). This procedure aimed to confirm that no articles were excluded inappropriately. The re-evaluation resulted in 100% agreement, confirming the reliability of the exclusion decisions.

If there was no abstract available, the full text article was checked for eligibility. Supplementary sources were obtained from reference lists and direct communication with authors.

After screening, 170 publications were included for full text reading (online supplemental appendix 1).

### Data extraction and analysis

The identified publications were classified according to the developmental framework by Selb *et al*^3^ and looked at reports on possibilities to use developed CS in practice and their generalisability to other settings and populations. Classification was based on the primary objective and methodology reported in each publication. Studies describing systematic reviews, patient perspective studies, expert surveys and protocol/discussion papers were assigned to the preparatory phase. Publications reporting on international consensus conferences were assigned to Phase I. Validation studies, implementation studies and studies evaluating the applicability of an ICF CS in specific professional groups, settings or countries were assigned to Phase II (figure 1). When publications addressed more than one developmental phase, they were linked to all relevant phases and were considered in each corresponding section of the analysis.

Data extraction was performed using a structured extraction framework aligned with the methodological components of ICF CS development. Extracted data included study type, developmental phase, study methodology, sample size, participant characteristics, professional disciplines involved, country and WHO region, consensus procedures, validation activities, implementation activities, publication year and journal.

To provide insight into the practical applicability and generalisability of developed CSs, information was extracted regarding the populations involved, professional groups represented, geographical distribution of participants, and the extent to which studies included stakeholders from multiple WHO regions. An overview of all included and linked studies can be found in online supplemental appendixs 1, 2.

## Results

### Study selection and characteristics

For 74% of the selected publications, research was performed in European countries. Approximately 30% were published in two journals: *Disability and Rehabilitation* and *Journal of Rehabilitation Medicine*. Three peaks in publication years were observed over the period 2001–2022: (1) in 2004, there were 17 publications; 13% of all publications, (2) in 2010–2011, there were 24 publications; 18% and (3) in 2013–2014, there were 24 publications; 18%. The number of publications between 2010 and 2014 could be attributed to 43% of all publications.

A total of 34 CSs were addressed in the included publications and covered various health conditions: 32 publications (23%) focused on cardiovascular and respiratory conditions, 30 (23%) on musculoskeletal conditions, 23 (17%) on neurological conditions, 20 (15%) on diverse situations (ie, vocational rehabilitation and generic and disability sets), 11 (8%) on other health conditions (eg, hearing loss and autism spectrum), 11 (8%) on cancer and 7 (5%) on mental health (online supplemental appendix 1).

For the available comprehensive CS, an average of 97 categories were included (SD 29; min 36 to max 159). For the brief CS, there was an average of 26 categories (SD 12; min 8 to max 60) (table 1).

**Table 1:** Overview of the number of categories included within the comprehensive and brief CS

	Comprehensive CS	Brief CS
	Mean; SD (range)	Mean; SD (range)
Environmental factors	26.6; 8.4 (10–48)	7.0; 5.0 (2–23)
Activity & participation	33.8; 14 (7–61)	8.9; 4.3 (3–19)
Body structures	7.7; 7.7 (0–33)	2.4; 2.2 (0–9)
Body functions	28.9; 13.8 (8–63)	8.4; 3.8 (2–18)
Total	97.1; 29.4 (36–159)	26.6; 12.1 (8–60)

CS, Core Set.

On average, both the comprehensive and brief CS exhibited a balanced distribution of included categories across body functions (30%), activity and participation (35%) and environmental factors (27%). While body structures represented a minority, they accounted for 8% in the comprehensive CS and 2% in the brief CS (table 1). When examining the methods by which the development of the CS was funded, a wide variety of sources were observed. The majority of CS developments were supported by European grants, national funding sources from the Ministry of Healthcare or Research Councils, or through financial contributions from charity foundations and advocacy groups.

### Methodologies used in the preparatory phase of the development of an ICF CS

Of all the included publications, 13 (8%) were about proposing the intention and study protocol to develop a CS as provided in invitation papers, 12 (7%) were systematic reviews, 28 (17%) were patient surveys, 12 (7%) were general expert surveys, 28 (17%) were consensus conferences and 63 (38%) were about validation in implementation studies. In addition, there were 13 (8%) studies that covered the entire development process (figure 3).

**Figure 3:**
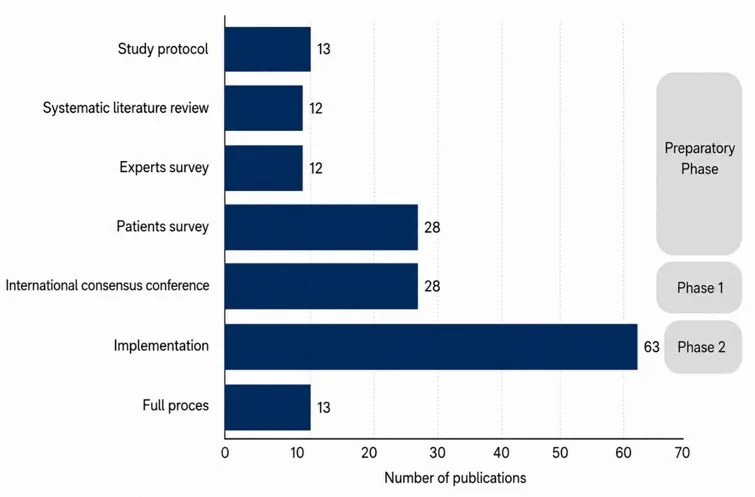
An overview of the number of included studies by development stage.

#### Study protocol/discussion papers

In 13 cases, study protocol papers were published to explain the outline of the methodology and/or an invitation to collaborate in the further development of the CS (figure 3). This was for example the case for the CS vision loss, hearing loss and deafblindness.^14–16^

#### Literature reviews

In 14 cases, a systematic literature review was conducted. The methodologies that were used were diverse. Most systematic reviews were performed to identify the most often studied areas and outcome measures in a specific population, as this would indicate the importance of ICF categories that should be included in a CS. The number of included publications ranged from 50 to 640 publications (mean 210.5). Methodological approaches differed according to the target population and CS under development. Some reviews focused on identifying commonly reported functioning problems and health-related concepts, whereas others primarily examined outcome measures or combined both approaches. Across all reviews, identified concepts were linked to ICF categories using the ICF linking rules proposed by Cieza *et al*.^4^ Inclusion and exclusion criteria varied between reviews but generally focused on adult populations, English-language publications and studies involving the target condition without substantial comorbidities (table 2).

**Table 2:** Characteristics of the systematic reviews

ICF core set	Inclusion criteria	Exclusion criteria	Used databases	Sample size (n)
Cerebral palsy^34^	Publication date: 2000–2017; age >18 years; n>10; observational and experimental study design; describing only CP; reporting about outcome and functioning	Meta-analyses; reviews; case study; qualitative studies; study protocols; evaluation of services or surgery or complications	Embase; Medline; Web of Science; PsycINFO; CINAHL; Cochrane; Google Scholar	N=274
Schizophrenia^35^	Publication date: 2008–2012; age >18 years; studies in English; human participants; peer-reviewed articles	Meta-analyses; reviews articles; psychometric studies; preventive studies	CINAHL; Medline; PsycINFO	N=348
Bipolar disorders^36^	Publication date: 2000–2006; age >18 years; n>10; English-language, diagnosed with BD type 1 or 2; randomised controlled, randomised clinical, clinical controlled, cross-sectional, longitudinal observational or qualitative study designs.	No data	Medline; PsycINFO	N=109
Hearing loss^37 38^	Publication date: 2002–2007; age >18 years; n>10; observational studies; focus on target group (condition must relate to HL); HL from mild until profound; use of oral communication as primary mode; controlled experimental studies; economic evaluation	Non-human populations; age <18 years or mixed age groups; psychometric evaluation studies; case reports	Medline; CINAHL; Embase; PsycINFO; AMED; ERIC; Cochrane; PubMed	N=659
Vision loss^39^	Age ≥18 years; n≥10; English-language studies using systematic reviews, observational, prospective longitudinal, RCT, cross-sectional, cohort or psychometric designs; VL ranging from mild to blindness; any vision condition not attributable to another medical condition.	Non-human populations; publication date <2001; prevalence and intervention studies; visual impairment because of a comorbid condition	PubMed; Embase; CINAHL; PsycINFO; Web of Science	N=369
Deafblind^40^	Publication date: 2011–2022; aged >18 years; deafblindness; any country/language	Study designs: reviews, focused on genetics, prevention, laboratory experiments, study protocols, letters, editorials and comments; n<5	Medline; Embase; CINAHL; PsycINFO; Web of Science; Global Health; ERIC; Google Scholar; Örebro	N=147
Sleep^41^	Study design: study included measures and questionnaire	No data	Medline; Embase; PsycINFO	N=115
Breast cancer^42^	Publication date: 1991–2000; studies in English	Study design: reviews, secondary analyses, primary prevention, psychometric, mode of action and heterogeneous population studies	Medline	N=734
Stroke^43^	Age >18 years; study designs: outcome measures and observation; mixed population; studies in English	Study design: intervention studies	Web of Science; AMED; PsycINFO; CINAHL; Medline	N=212
Geriatric patients^44^	Publication date: 2007–2017; age >75 years; study designs: RCT, CCT, cross-sectional, observational and qualitative research	Study designs: study protocols, case, psychometric, prevention studies, phase II clinical trials, systematic reviews, meta-analyses	CINAHL; Embase; PsycINFO; PubMed; Scopus	N=68
Vocational rehabilitation^45^	Publication date: 2001−2016; ICF or “International Classification of Functioning, Disability and Health” mentioned in the title or abstract; ICF used in the field of vocational rehabilitation research; peer-reviewed original papers and reviews; abstract available; study populations: working age adults	Papers only mentioning ICF in the abstract background or discussion; only mentioning ICIDH or ICIDH-2. Study designs: overviews, editorials, comments, theoretical papers, text and opinion papers, theses/dissertations, books and papers on ICF	PubMed; Embase; Scopus; CINAHL; PsycINFO; SweMed; PEDro	N=50
ADHD^46^	Publication date: 1982 onwards; participants of any age with a primary diagnosis of ADHD according to DSM, ICD, clinical assessment, diagnostic instruments; study designs: all types of outcome (self-report, proxy report or observational/performance data) and studies of any design that present original data; studies in English	Citations of an excluded publication type (eg, books, dissertation abstracts, published in a language other than English), the main outcome of the studies was not ability or disability in ADHD, use of an excluded design, or including participants from an excluded population (ie, not having ADHD diagnosis)	Medline; PubMed; PsycINFO; ERIC; CINAHL	N=226
Autism spectrum disorder^47^	Publication date: 1992 or later; population: participants of any age with any ASD diagnosis; study designs: studies of any design that present original data and all types of outcome; studies in English	No data	Medline; PubMed; PsycINFO; ERIC; CINAHL	N=154
Amputation lower limb^48^	Publication date: 1996–2010; age >18 years; study designs: observational studies, RCT	Age <18 years; animal studies; study designs: reviews, comment, correspondence and case reports	Medline; Embase; PsycINFO; CINAHL	N=113

ICF, International Classification of Functioning, Disability and Health.

#### Patient perspective studies

Patient perspective studies represented an important component of the preparatory phase and were identified for multiple ICF CSs (table 3). These studies aimed to explore patients’ experiences of functioning, disability and participation to ensure that the resulting CSs reflected lived experiences.

**Table 3:** Characteristics of the patient perspective

ICF core set	Sample size	Countries	Methodology
Ankylosing spondylitis^49 50^	N=30 N=111	1 country (The Netherlands) 2 countries (The Netherlands & Germany)	Focus groups Structured interviews with the extended ICF checklist
Low back pain^51^	N=118	1 country (Norwegian)	The patients reported their health problems in the WHODAS II and ODI
Arthritis^52^	N=21	1 country (Austria)	Interviews
Cerebral palsy^53 54^	N=38 N=20	Dutch speaking participants 1 country (Colombia)	Focus group (n=31) and individual interviews (n=7) semi-structured interviews(10 caregivers and 10 children)
Traumatic brain injury^55^	N=261	1 country (Italy)	Data collection by the Case Record Form
Multiple sclerosis^56^	N=113	1 country (Finland)	Group followed for 2 years, data collected with the Canadian Occupational Performance Measure
Bipolar disorders^57^	N=15	1 country (Spain)	Semi-structured interviews
Vertigo^58^	N=12	1 country (Germany)	Interviews
Hearing loss^16^	N=36	2 countries, 2 WHO regions (South Africa and the Netherlands)	Focus groups
Deafblindness^59^	N=72	10 countries, all WHO regions (Canada, Mexico, Australia, Spain, UK, France, Zambia, Egypt, India, Nepal)	Focus groups (9) Individual interviews (n=26)
Sleep^60 61^	N=27 N=99	Not clear (3 different sleep centres) 1 country (Swiss)	Focus groups Interviews and case records collected by using the ICF checklist
Head and neck cancer ^62–65^	N=18 N=300 N=145	1 country (Germany) 1 country (Germany) 4 countries, 1 WHO area (Greece, Germany, Poland, Turkey)	Interviews Own created checklist based on the brief core set Patient interviews according to ICF checklist and EORTC-QLQ-C30.
Breast cancer^66^	N=34 N=85	1 country (Ireland) 1 country (Australian)	Focus groups Interviews
Brain tumour^67^	N=106	1 country (Australian)	Interview
Stroke^68 69^	N=60 N=221	1 country (Germany) 1 country (Finland)	Focus groups semi-structured interview
Obesity^70^	N=51,	1 country (Italy)	Data collected by using diagnostic therapeutic protocol
Diabetes mellitus^71^	N=40	1 country (Germany)	Digitally recorded focus groups
Geriatric patients^72^	N=150	1 country (Germany)	Interview
ADHD^73 74^	N=76 N=112	5 countries, 5 WHO regions (Brazil, India, Saudi Arabia, South Africa and Sweden) 8 countries, 4 WHO regions (Denmark, Germany, Italy, Sweden, Portugal, Saudi Arabia, India and Taiwan)	Focus groups and semi-structured interviews. Extended ICF-CY checklist
Autism spectrum disorder^75^	N=122	10 countries, 4 WHO regions (Argentina, Brazil, Denmark, Germany, Portugal, Greece, Italy, Sweden, Japan, Saudi Arabia)	Interviews
Amputation lower limb^76^	N=83	2 countries, 1 WHO region (China & Australia)	Face-to-face interviews

ICF, International Classification of Functioning, Disability and Health.

In preparation for the development of various ICF CSs, multiple patient surveys were conducted to gather insights into functioning and health-related challenges across different conditions. These studies used diverse methodologies, including focus group meetings, structured interviews and standardised checklists, to ensure comprehensive data collection. Sample sizes varied from 12 to 300 participants, with research spanning from 1 to 10 countries, covering multiple WHO regions. International representation varied considerably. Most patient perspective studies were performed in only one country or WHO region. Only a limited number included participants from multiple WHO regions. In three studies, there was an involvement of two WHO regions, and always in combination with Europe. The majority were held in one or two specific countries, such as South Africa and the Netherlands for the CS hearing loss. In this study, cultural differences were considered.^17^ Only one study on systemic sclerosis was found, originating from France, which had a strong focus on environmental and personal factors that were meaningful to patients.^18^

##### Focus groups

Focus groups were among the most frequently used methodologies. The number of participants ranged from 17 to 76 and participants were recruited through professional networks, patient organisations, or external recruitment strategies. All concepts identified during the discussions were linked to ICF categories using the linking rules proposed by Cieza *et al*.^4 5^

##### Interviews

Individual and semi-structured interviews were widely used across different health conditions, including bipolar disorder, vertigo, head and neck cancer, breast cancer, stroke, and autism spectrum disorder. Sample sizes ranged from 12 to 300 participants. Several studies complemented interviews with standardised instruments such as the ICF Checklist, EORTC-QLQ-C30 or disease-specific assessments.

##### Questionnaires

Regarding studies in which questionnaires were used, the number of participants ranged from 85 to 118, with an open-ended questionnaire used in all cases and often supplemented by validated instruments such as the WHODAS II, ODI, Canadian Occupational Performance Measure, and extended ICF checklists to document patient experiences.

### Expert surveys

In order to map the experts’ perspectives, an internet-based questionnaire was conducted in almost all studies. The experts were required to meet specific inclusion criteria, including having a professional background and involvement with patients, having between two and 5 years of work experience, and the ability to respond in English. The questionnaire consisted of two parts, a demographic and an open-ended part, to gain insights into the functioning of the target group. The expert group included individuals from various disciplines and represented all WHO regions. However, the distribution across WHO regions was not always balanced, often favouring the European region (table 4).

**Table 4:** Characteristics of the perspective of experts

ICF core set	Sample size	Sample composition	Recruitment	Countries	Method
Ankylosing spondylitis^77^	N=74	Rheumatologists, nurses, physiotherapists, occupational therapists, psychologists, rehabilitation physicians and social workers	Mailing to the work group assessment of Spondylosing Arthritis International Society and national and international associations of rheumatology.	Countries not specified, 5 WHO regions	Delphi method
Cerebral palsy Focus on children^78^ Focus on adults^79^	N=193 N=125	Therapist, physician, educator, rehabilitation nurse and social worker Physicians, physiotherapist, occupational therapist, speech therapist, nurse, social worker, researchers	Mailing to organisations, invitation letter on the website, authors who published an article in a paediatric journal mailing to organisations, research groups, authors involved in systematic review, conferences, snowball sampling	Countries not specified, all WHO regions included Countries not specified, all WHO regions	Online survey
Schizophrenia^80^	N=1722	Diverse group of professionals, (psychiatrists and psychologists representing 65.14% of the sample, the rest is not specified)	Experts selected from literature and contacted by mail	27 countries (not specified), all WHO regions included	Online survey
Hearing loss^81^	N=63	Audiologist, physicians, engineer, speech pathologist, psychologist, social worker, teacher, hearing aid dispenser	International organisations and publishers were contacted by mail	All WHO regions included	Internet-based survey
Deafblind^82^	N=105	Teacher, intervenor, rehabilitation professional, administrators, social worker, psychologist, audiologist, physician, physiotherapists, speech pathologist, low vision therapist, volunteers/parents of a child, orientation and mobility therapist, occupational therapist, optometrist, personal support worker, academician	Social media, international organisations, snowballing approach	All WHO regions included	Online survey
Head and neck cancer^83^	N=103	Physicians, dentists, psychologists, physiotherapists, speech therapists, nurses and social workers	Contacted worldwide organisations engaged in the treatment of HNC	27 countries, all WHO regions included	Internet-based survey
Geriatric patients^84 85^	N=40 N=63	Physicians, nurse, physical therapist, occupational therapist, speech therapist, psychologist, nutrition therapist Physicians, nurses, physical therapist, occupational therapist, speech therapist, psychologist	3 university hospitals in Germany, Switzerland and Austria Participants recruited by regional networks of GPs	European region 1 country (Germany)	Focus groups Delphi method Internet survey
Vocational rehabilitation^86^	N=23	Occupational therapist, physical therapist, psychologist, medicine and social work	Literature, (inter)national (non)-professional organisations, internet search, informal network, editorial board of journals, referred peers.	All WHO regions	Formal decision-making and consensus-based process.
ADHD^87^	N=174	Coach, counsellor, nurse, occupational therapist, physician, physiotherapist, psychologist, psychotherapist, social worker, special educator, speech therapist	Internet search, key opinion leaders, own professional network, snowball sampling	All WHO regions	Email-based survey
Autism spectrum disorder^88^	N=245	Coaches, nurses, occupational therapists, physicians, physical therapist, psychologist, social worker, special educator and speech therapist	Experts, universities, clinics	All WHO regions	Email-based survey.
Amputation lower limb^76^	N=132	Prosthetist, physician, physiotherapist, surgeon, occupational therapist, nurse, prosthesis manufacturer, psychologist, recreational therapist	Attending the Prosthetics and Orthotics International Conference in 2013	5 WHO regions	Face-to-face questionnaire (n=25) Online survey (n=107)

ICF, International Classification of Functioning, Disability and Health.

#### Professional disciplines

The expert groups were multidisciplinary and included healthcare professionals from a wide range of backgrounds. Physical therapists (27%), medical specialists (18%) and occupational therapists (15%) were the most frequently represented professions. Additional disciplines included psychologists, speech and language therapists, psychiatrists, social workers, nurses, teachers, researchers and rehabilitation professionals. In 18% of studies, experts were recruited as heterogeneous multidisciplinary groups (table 5).

**Table 5:** Overview of occupations that were involved in compiling the professional perspective

Occupations (n=32)	N (%)
Physical therapists	9 (27%)
Medical specialists	6 (18%)
Occupational therapists	5 (15%)
Psychologists	3 (9%)
Speech and language therapists	2 (6%)
Psychiatrists	2 (6%)
Mixed groups	6 (18%)

### Methodologies used in phase I

A total of 28 publications reported on the consensus phase (Phase I) of ICF CS development. During this phase, findings from the preparatory studies were integrated and translated into a first version of the comprehensive and brief ICF CSs through structured consensus procedures (table 6).

**Table 6:** Characteristics of the international consensus conferences

ICF core set	Sample size	Sample frame	Countries included	Methodology
Chronic pain^89^	N=30	Rheumatologists, psychiatrists, physical therapists, psychologist, occupational therapist and social worker	10 different countries	Delphi method
Osteoporosis^90^	N=15	Physicians and physical therapists	7 different countries	Delphi method
Low back pain^91^	N=18	Physicians, occupational therapists and physiotherapist	15 different countries	Delphi method
Arthritis^92^	N=17	Physicians and physical therapists	7 different countries	Formal decision-making and consensus process
Cerebral palsy Children^21^ Adults^93^	N=26 N=25	Therapists, physicians, teacher and 1 parent Patient, parent, orthopaedic surgeon, physical therapist, researcher, occupational therapist, speech therapist, endocrinologist, nurse, gerontologist, psychologist and physician	All WHO regions All WHO regions	3-day physical workshop online decision-making by Zoom (4 weeks, weekly sessions of 3 hours)
Spinal cord injury^94^	N=20	Physiatrists, neurosurgeons, occupational therapists, psychologists, physical therapists and social workers.	1 country (Taiwan)	Delphi method
Multiple sclerosis^20^	N=21	Physicians, physical therapists, occupational therapists, nurses, psychologists, speech therapist, social workers and the chief executive ofﬁcer of an MS Society Centre	16 different countries, all WHO regions included	Physical, iterative decision-making and consensus process
Bipolar disorder^95^	N=21	Psychiatrists, psychologists and nurse	15 from Europe and 6 from 3 WHO regions	Physical consensus conference
Depression^96^	N=20	Psychiatrists, physicians, psychologists, physical therapists, nurse and an occupational therapist	8 countries	Formal decision-making and consensus (ranking exercise)
Vertigo^97^	N=27	Physicians, physical therapists, occupational therapist, sports scientist, psychologist, expert in medical physics and vestibular disorders.	No data	Consensus conference
Hearing loss^8^	N=21	Patients, audiology, speech-language pathology, rehabilitation sciences, audiological medicine, psychology, otorhinolaryngology, hearing device specialty/technical audiology	14 countries, all WHO regions	Consensus conference
Sleep^98^	N=26	No data	22 countries	Consensus conference using Delphi method
Head and neck cancer^19^	N=21	Physicians, psychologists, physiotherapists, nurse and social worker	12 countries, all WHO regions	Consensus conference
Breast cancer^99^	N=19	Physicians, physiotherapists, occupational therapists, psychologist, epidemiologist, nurse	5 different countries	Consensus conference
Brain tumour^100^	N=29	Paediatric neurosurgeons, paediatric oncologists, paediatric neurologist, nurses, psychologists, social workers, physical therapists, occupational therapists, speech therapist and special needs teachers	One country (China)	Delphi method by mail
Stroke^101^	N=36	Physicians, physical therapists, psychologists, social worker and sociologist	12 different countries	Delphi method
Obesity^102^	N=21	Physicians and physical therapists	8 different countries	Delphi method
Diabetes mellitus^103^	N=15	Physicians, physical therapists, epidemiologist and 1 social worker	9 different countries	Delphi method
Obstructive pulmonary disease^104^	N=17	Physicians, physical therapists, psychologist and nurse	8 different countries	Consensus conference
Cardiopulmonary conditions^105^	N=22	Physicians, therapists (not defined), nurses and a social worker	3 countries, European WHO region	Consensus conference
Chronic ischaemic heart disease^106^	N=16	Physicians, physical therapists and epidemiologists	7 different countries	Consensus conference
Geriatric patient^107^	N=15	Physicians, therapists (not defined) and nurses	3 countries, only European Region	Consensus conference
Burn injuries^108^	N=30	Plastic surgeons, physiatrists, dermatologist, psychiatrist, physical therapists, occupational therapists, speech therapist, psychologists, social workers, public health scholars, nurses and the director of the Taiwan Vocational Rehabilitation Association	No data	Online Delphi

ICF, International Classification of Functioning, Disability and Health.

#### Delphi methods versus face-to-face consensus conferences

The majority of early CSs were developed through face-to-face international consensus conferences, during which preparatory evidence was discussed and voting procedures were used to determine the inclusion and exclusion of ICF categories. Several studies complemented these meetings with structured discussion rounds, ranking exercises or formal decision-making procedures.

In contrast, more recently developed CSs increasingly incorporated Delphi methodologies or fully online consensus procedures. These approaches allowed experts from different countries to participate remotely and facilitated iterative feedback over multiple rounds. Hybrid approaches combining Delphi surveys with virtual meetings were also observed, particularly in more recent developments such as the adult cerebral palsy CS.

Overall, consensus procedures evolved from predominantly face-to-face conferences towards hybrid and Delphi-based approaches, while patient participation remained limited throughout most consensus processes.

#### Number of experts and composition of consensus groups

The number of participants involved in consensus activities ranged from 15 to 36 experts, with an average of 22.2 participants per consensus process. Physicians were the most frequently represented professional group (70% of studies), followed by physical therapists (65%) and psychologists (39%). The composition of expert panels varied according to the target population and healthcare context of the CS under development. In some more recently developed CSs, additional stakeholders such as researchers, educators, patient representatives, caregivers and representatives from patient organisations were involved. However, patient participation during the consensus phase remained limited and was only explicitly reported for a small number of CSs, such as hearing loss and cerebral palsy.

Consensus groups were multidisciplinary and generally consisted of healthcare professionals with expertise in the target condition. Frequently represented professions included physicians, physical therapists, occupational therapists, psychologists, nurses, speech and language therapists, and social workers.

#### International representation

International representation was a key objective of most consensus processes. Consensus groups included experts from between 1 and 22 countries. However, the degree of international representation varied considerably across CSs. For several studies, only the number of participating countries was reported, making it difficult to determine whether all WHO regions were represented.

Confirmed representation from all WHO regions was reported for only a limited number of CSs, including hearing loss,^10^ head and neck cancer,^19^ multiple sclerosis^20^ and cerebral palsy.^21^ Other CSs relied primarily on participants from European countries or involved experts from a restricted number of regions. Overall, Europe remained the most consistently represented WHO region throughout the consensus phase.

### Studies in phase II

A total of 63 publications focused on the validation and implementation phase (Phase II) of ICF CS development, making this the most frequently reported phase in the included literature (figure 3). These studies aimed to evaluate the applicability, relevance and feasibility of existing ICF CSs across different healthcare settings, professional groups and geographical regions.

Validation activities were not equally distributed across all ICF CSs. Most validation studies focused on CSs developed for arthritis, stroke and obstructive pulmonary disease. Together, these conditions accounted for approximately 42% of all validation and implementation studies identified in the review.

#### Validation methodologie

The most frequently applied validation methodology was a (electronic) multi-round Delphi process. In the first round, participants provided open-ended responses that were linked to ICF categories. In the second round, participants assessed agreement on the chosen categories, taking into account their representation of a patient’s ability or disability. In addition, in several studies, the CS was compared with often used outcome measures that were found in the literature.^22^

#### Regional and international validation

Many studies investigated the transferability and applicability of CSs across different countries and cultural settings. Validation studies were conducted in various regions, including Malaysia,^23^ Taiwan,^24^ China^25^ and several European countries. Surveys were frequently made available in multiple languages^26^ to facilitate international participation and improve cultural relevance.

Almost all validation studies included participants from multiple WHO regions, reflecting a stronger international orientation than was observed during earlier developmental phases. Nevertheless, regional differences in representation remained evident across studies.

#### Professional perspectives

A major focus of the validation phase was assessing the applicability of developed ICF CSs across different healthcare professions. Validation studies were conducted from the perspectives of physical therapists,^27 28^ occupational therapists,^29^ speech and language therapists^30^ and other professional groups. Recruitment of professionals was typically achieved through international professional federations, literature searches, professional networks, personal recommendations and social media platforms such as LinkedIn. This professional validation process aimed to determine whether the selected ICF categories adequately reflected the information required by different disciplines involved in patient care.

### Methodological findings

Across all phases of ICF CS development, several recurring methodological trends emerged. First, healthcare professionals were consistently involved throughout the entire development process, including the preparatory, consensus and validation phases. In contrast, patient involvement was primarily concentrated in the preparatory phase through focus groups, interviews and questionnaires. Although several recent ICF CS developments have increased patient participation, direct involvement of patients during consensus conferences remained limited.

Second, considerable variation was observed in the geographical representation of stakeholders. While international participation was an explicit objective of many studies, Europe was consistently the most represented WHO region across all developmental phases. Patient perspective studies were frequently conducted within a single country, whereas expert surveys, consensus activities and validation studies more often involved participants from multiple countries and WHO regions. However, balanced representation across all WHO regions was only achieved in a limited number of CS developments.

Third, substantial methodological diversity was evident throughout the development process. Preparatory studies used a wide range of methods, including systematic reviews, focus groups, interviews, questionnaires and standardised assessments. Similarly, consensus procedures varied from traditional face-to-face conferences to Delphi studies, online surveys and hybrid consensus approaches. Validation studies also differed considerably with respect to stakeholder groups, recruitment strategies and methods used to assess content validity and applicability.

A fourth observation was the evolution of development methodologies over time. Earlier CS developments relied predominantly on face-to-face consensus conferences, smaller expert panels and limited international participation. More recent developments increasingly incorporated electronic Delphi methodologies, virtual consensus meetings, multilingual surveys and broader international recruitment strategies, facilitating greater stakeholder involvement and global participation.

Finally, important gaps in validation and implementation activities were identified. Although validation and implementation studies represented the largest category of included publications, their distribution across CSs was uneven. A substantial proportion of studies focused on a limited number of conditions, particularly arthritis, stroke and obstructive pulmonary disease. Furthermore, many validation studies examined the applicability of CSs from a single professional or regional perspective rather than using a comprehensive multi-stakeholder approach. As a result, evidence regarding the global applicability and implementation of several CSs remains limited.

Overall findings suggest that future ICF CS developments would benefit from broader patient involvement across all developmental phases, more balanced geographical representation and greater methodological standardisation during validation and implementation activities. These improvements may strengthen the international relevance, applicability and uptake of future ICF CSs.

## Discussion

This systematic scoping review offers a comprehensive overview of the methodologies used for the development of previous ICF CS from 2001 to 2024. We expand on previous reviews by examining all developmental phases of ICF CS development, including the preparatory, consensus and validation/implementation phases. Our findings demonstrate substantial methodological variation across all phases of development, particularly with respect to stakeholder involvement, geographical representation, consensus procedures and validation approaches. While most CS developments incorporated the developmental phases proposed by Selb *et al*,^3^ considerable variation was observed in how these phases were operationalised.

We found that most of the included studies were originated from Europe and the USA. Initially, a select group of researchers from Europe and the USA led the creation of the CS. Although international participation increased over time, geographical representation remained uneven across developmental phases, with Europe consistently being the most represented WHO region.

The findings also suggest that ICF CS development methodologies have evolved substantially over time. Early CS developments, particularly those published between 2004 and 2010, relied predominantly on face-to-face international consensus conferences involving relatively small multidisciplinary expert groups. Patient involvement during these developments was often limited to preparatory studies and international representation was not always achieved across all WHO regions. In contrast, more recently developed CSs increasingly adopted electronic and hybrid methodologies, including online expert surveys, electronic Delphi procedures, virtual consensus meetings and multilingual data collection strategies. Examples include the development of the CSs for hearing loss, deafblindness, ADHD, autism spectrum disorder and cerebral palsy, which demonstrated broader stakeholder involvement and greater efforts to achieve global representation. These developments appear to facilitate participation from geographically dispersed stakeholders and may contribute to the international applicability of future CSs.

According to the guidelines provided by Selb *et al,*^3^ a CS needs to include the typical spectrum of problems in functioning experienced by patients. We found an average of 97 categories in the comprehensive CS and an average of 27 categories in the brief CS. The focus was mainly on ‘Body Functions’, ‘Activities and Participation’ and ‘Environment’ and less on ‘Body Structures’. We noticed that five comprehensive CS and seven brief CS did not include a single item from the component ‘Body Structures’. This finding indicates that Body Structures received less attention than the other ICF components. However, the reasons underlying this pattern could not be determined from the available data and therefore warrant further investigation. Most CS developments incorporated the developmental phases described by Selb *et al*,^3^ although important variation was observed in the way these phases were implemented. Study protocols were often not provided and whereas a global approach is deemed essential to ensure cultural validity, several studies refrained from using this approach, for example, in the CS cardiopulmonary, musculoskeletal and neurological conditions in the acute and post-acute setting. This may be due to feasibility problems. The ICF linking rules provide a structured method to link health-related information to the ICF. Key steps included understanding the ICF framework, identifying the main and additional concepts, considering perspectives and categorising response options where the identified concepts to the most precise ICF categories were mapped. These refinements ensured transparent and reliable data linkage for research and health decision-making.

During the international conferences, in several cases, one consensus conference was used instead of separate conferences for each condition group. In such situations, it is not easy to gather experts who have sufficient in-depth knowledge and experience of all the disorders targeted by a certain CS, reducing the quality of the outcomes.

Experts were consistently involved in CS development, with varying approaches based on the specific CS being developed. Some used a homogeneous approach, focusing on a specific professional disciplines, while others employed a heterogeneous approach, involving various experts that worked with the target group. Multidisciplinary stakeholder involvement may provide broader perspectives on functioning and disability and may therefore contribute to a more comprehensive understanding of the needs of the target population. Each professional contributes unique insights based on their specific role and interaction with the patient, enabling a more holistic understanding of needs and care dynamics. This diversity enhances the relevance, applicability and completeness of the developed solution. Therefore, selecting a heterogeneous group from the outset should be actively pursued, although this may be influenced by the type and scope of funding supporting the research.

Patients were frequently involved during the preparatory phase, primarily through focus groups, interviews and questionnaires. However, patiënt participation was less frequently reported during consensus and validation activities. Given the increasing recognition of patients as experts by experience, future CS developments may benefit from exploring opportunities for patient involvement throughout all phases of the development process. In the preliminary versions of the CS, participants were typically professional experts in this phase. However, patients are nowadays considered experts by experience and can provide significant added value in determining which categories should be included in the CS.^31 32^ An important finding of this review was the substantial variation observed in validation and implementation methodologies. Although validation and implementation studies represented the largest category of included publications, these activities were not equally distributed across all CSs. Furthermore, many validation studies focused on a single professional group or geographical region, potentially limiting conclusions regarding broader applicability.

A key finding of this review was the considerable methodological diversity observed across all phases of CS development. Preparatory studies employed systematic reviews, interviews, focus groups, questionnaires and standardised assessments. Consensus procedures ranged from face-to-face international conferences to Delphi techniques and hybrid online approaches. Similarly, validation studies differed in stakeholder groups, recruitment strategies, geographical scope and methodological design. While this flexibility may facilitate adaptation to different health conditions and contexts, it may also reduce comparability across CS developments.

As the guidelines by Selb lack specific information how to validate a CS, we recommend to involve diverse stakeholders, including patients of different ages and WHO regions, as well as professionals from various healthcare disciplines to have a broad check, based on consensus, to determine if the most important categories are actually present in the CS. Methods recommended are Delphi rounds where stakeholders can indicate the importance for every category and indicate whether categories are missing. With regard to implementation, the practical application of the CS should be tested. As a CS is merely an overview of important ICF categories for a certain condition, it is not a measurement instrument. In order to use the ICF CS as an overview, the CS could be implemented into healthcare policy as a check to see if patients are treated according to their CS specific holistic views. In addition, a CS could potentially be used as a blueprint for constructs that could be measured, for example, to study effects of treatment. In that case, and as part of an implementation study, measurement instruments or patient-reported outcome measures should be chosen and checked against the ICF categories of the available CS. Current Core Outcome Measurement methodology may provide additional guidelines on how to reach consensus on which instruments to use. After the choice of instruments, the actual implementation could be studied using process evaluations or so called ‘problem analyses’. Examining the methodologies employed for its implementation, which tools were developed, and the creation of documentation forms and treatment guidelines to support and facilitate its integration into clinical practice.

Overall, this review underscores the importance of cultural aspects and a global mindset throughout the CS development process. Proper attention to the perspectives of both patients and professionals during the validation phase is essential for developing an effective and feasible CS. Usability should be assessed across different regions and professional groups, considering a holistic view, an international context and individuals’ beliefs, priorities and preferences for managing health, illness and disability. It is crucial to adopt an international mindset from the outset of development and to give it sufficient attention during the preliminary phase. Although validation studies often expanded to include various users and countries, they have been mostly approached from a single perspective and within a European context.

### Strengths & limitations

This research benefits from a robust methodological process, incorporating a systematic review that builds on data from previous work by Yen *et al*^6^ up to 2022. Unlike previous studies, our review encompasses all phases involved in creating a CS rather than focusing on specific phases. However, there are certain limitations to consider. Our systematic review relied primarily on the PubMed database and therefore may not have captured all methodological publications related to ICF CS development. While the official ICF CSs platform (https://www.icf-core-sets.org/) enabled us to verify the inclusion of all existing CSs, this does not necessarily guarantee that all publications describing preparatory studies, consensus procedures, validation activities or implementation work associated with those CSs were identified. To mitigate this risk, we supplemented the database search through reference screening and direct communication with authors. Nevertheless, methodological publications indexed exclusively in databases such as CINAHL, PsycINFO, Embase or Scopus may have been missed. Consequently, some methodological variations may not have been fully captured in this review.

## Conclusion

We conducted a systematic scoping review to investigate the past developmental methodologies of ICF CS. Our study contributes to understanding the different methodologies used. We thoroughly described and synthesised the different phases of the CS development process, the level of expert and patient involvement and the observed variation across different initiatives. Notably, the methodology of the included studies varied significantly, and there was limited representation of studies from outside Europe. To enhance the practical relevance of future CS, it is essential to carefully consider and integrate the perspectives of potential users, including patients and experts from all WHO regions. Based on the findings of this review, future ICF CS developments may benefit from three key considerations. First, broader patient involvement across developmental phases may improve relevance and acceptability. Second, more balanced representation across WHO regions may strengthen international applicability. Third, greater consistency in validation and implementation methodologies may facilitate comparison across CSs and support wider uptake in clinical practice. These recommendations should be interpreted as potential directions for future development rather than conclusions directly demonstrated by the included studies.

## Supplementary material

10.1136/bmjopen-2026-123955 online supplemental appendix 1

10.1136/bmjopen-2026-123955 online supplemental appendix 2

## Data Availability

Data are available upon reasonable request.
