# Supplementary material for: Methodologies used in the development of the international classification of functioning, disability and health core sets: a systematic scoping review
Source: BMJ Open. 2026 Oct 5;16(10):e123955. doi: 10.1136/bmjopen-2026-123955 (PMC13646977; doi:10.1136/bmjopen-2026-123955)
Supplement: Supplementary file 1 [file bmjopen-2026-123955.reviewer_comments.pdf]

PEER REVIEW HISTORY

BMJ Open publishes all reviews undertaken for accepted manuscripts. Reviewers are asked to complete a checklist review form and are provided with free text boxes to elaborate on their assessment. These free text comments are reproduced below.

ARTICLE DETAILS

Title (Provisional)

Methodologies used in the development of the International Classification of Functioning, Disability, and Health Core Sets: a systematic scoping review

Authors

Billiet, Lorenzo; De Baets, Stijn; Van de Velde, Dominique; van Nispen, R. M.A.; van der Aa, Hilde P.A.

VERSION 1 - REVIEW

**Reviewer**	**1**
**Name**	**Shiota, Shigehito**
**Affiliation**	**Hiroshima University Hospital, Department of**
**Rehabilitation**	
**Date**	**08-Jul-2026**
**COI**	**None**

Overall comments

This manuscript addresses an important methodological topic: how ICF Core Sets have been developed across different health conditions and developmental phases. A review of this topic could provide valuable guidance for future ICF Core Set development, particularly regarding methodological transparency, patient and expert involvement, international representation, validation, and implementation.

However, in its current form, the manuscript requires substantial revision. The main concerns relate to the clarity of the study rationale, reproducibility of the search strategy, specificity of the eligibility criteria, transparency of the study selection process, alignment between the stated objective and the data extraction framework, organization of the Results section, and consistency across the Abstract, Results, Figures, Tables, Appendices, and References. The Discussion and Conclusion should also be revised after the Methods and Results have been clarified, as several interpretations currently appear stronger than what is directly supported by the reported findings.

I therefore recommend major revision.

Major comments

Major comment 1. Rationale and novelty of the review

The rationale for conducting this review could be strengthened. Although the authors cite previous reviews and state that existing guidelines are insufficiently specific, the manuscript does not clearly articulate the remaining knowledge gap. It would be helpful to explain more explicitly why previous reviews are insufficient, what methodological questions remain unanswered, and how the present review uniquely addresses these gaps. This would strengthen the novelty and significance of the study.

Major comment 2. Search strategy and reliance on PubMed

Although the authors acknowledge the limitation of relying primarily on PubMed, the justification provided mainly focuses on whether all existing ICF Core Sets were identified. However, the aim of this review is not merely to identify ICF Core Sets, but to review, classify, and analyze the methodologies and techniques used in their development. Therefore, the key issue is whether all relevant publications describing the development methodology of each Core Set were sufficiently identified.

The authors should clarify this distinction and discuss more explicitly how the PubMed-based search strategy may have affected the completeness of the methodological analysis. The limitations section should also be revised to distinguish between identifying existing Core Sets and identifying all relevant publications needed to analyze their development methodologies.

Major comment 3. Full search strategy should be reported

The authors state that relevant publications were identified in PubMed using standardized MeSH terms combined with Boolean expressions. However, the specific MeSH terms, free-text terms, Boolean operators, and full PubMed search strategy are not reported. This substantially limits the reproducibility and transparency of the review, particularly because PubMed was used as the primary database.

Please provide the complete PubMed search strategy, including all search terms, Boolean operators, limits, and date of search, preferably in the Methods section or as supplementary material.

Major comment 4. Eligibility criteria and study selection process

The eligibility criteria and study selection process require substantial clarification. The criterion of "publications reporting on the development of Core Sets" is broad, and it is unclear which types of publications were eligible, such as protocol papers, preparatory studies, consensus conference reports, validation studies, implementation studies, related methodological papers, or studies on ICF-based assessment sets and other ICF-derived tools. This lack of specificity limits the transparency and reproducibility of the study selection process.

Moreover, Figure 2 is internally inconsistent: 507 screened records minus 341 excluded records would leave 166 records, whereas 170 reports are shown as assessed for eligibility. The diagram also does not report exclusion reasons, duplicate removal, or whether any reports were excluded after full-text assessment. Please clarify the eligibility criteria and revise the PRISMA flow diagram with consistent numbers and clear exclusion reasons.

Major comment 5. Data extraction and analytical framework

The data extraction and analysis methods require further clarification. The authors state that the identified publications were linked to the developmental phases outlined by Selb et al.; however, it is unclear how this linkage was performed, who conducted the classification, whether multiple reviewers were involved, how disagreements were resolved, and how studies covering more than one developmental phase were handled.

In addition, the criteria used to assess the practical use and generalizability of developed Core Sets are not clearly described. Given that the aim of this review is to examine methodologies used in ICF Core Set development, the data extraction framework should be described in more detail and should be more explicitly aligned with the methodological components of Core Set development.

Major comment 6. Organization and presentation of the Results section

The Results section requires substantial reorganization and rewriting. At present, the presentation of results is difficult to follow, and the main findings are not clearly prioritized. The text often provides lengthy descriptive details, but it is not always clear how these findings relate to the study objective or to the analytical framework described in the Methods. In particular, the sections on expert perspectives, Phase I, and Phase II mix descriptions of study designs, participant characteristics, consensus methods, validation procedures, and implementation issues without a clear structure.

Please reorganize the Results section so that it is explicitly aligned with the study aim and methods. The main methodological findings should be presented in a logical order, preferably based on the predefined ICF Core Set development phases and/or clearly defined methodological categories. Figures and tables should serve as the primary means of presenting detailed data, while the main text should highlight and interpret the key findings rather than repeat or loosely summarize the tables. This restructuring would substantially improve the clarity, readability, and methodological contribution of the review.

For example, Figure 3 would be easier to interpret if exact numbers were shown and if the correspondence between categories and developmental phases was clearer.

Major comment 7. Consistency of units of analysis, figures, and tables

The Results section would benefit from clearer organization and greater consistency in the unit of analysis. The current section mixes results reported at the level of publications, studies, and Core Sets, making it difficult to interpret the findings. For example, the Abstract reports 36 Core Sets, whereas the Results section states that 34 Core Sets were addressed.

In addition, it is unclear whether the percentages by health condition are based on publications or Core Sets.

The references to tables also appear inconsistent. For example, Table 1 seems to present the number of ICF categories in comprehensive and brief Core Sets, rather than the distribution of health conditions, while Table 2 appears to describe characteristics of systematic reviews rather than the distribution of ICF components. Please clarify the unit of analysis throughout the Results section, ensure consistency between the Abstract, Results, Tables, Figures, and Appendices, and reorganize the results according to the methodological focus of the review.

Major comment 8. Alignment between Selb et al.'s framework and the presented classifications

Although the authors state that included publications were linked to the developmental phases outlined by Selb et al., the variables presented in the tables do not always appear to be directly derived from, or clearly mapped onto, Selb et al.'s methodological framework for ICF Core Set development. For example, Table 2 mainly summarizes general characteristics of systematic reviews, such as inclusion/exclusion criteria, databases, and sample size.

Please clarify how the table variables were selected and how they relate to the established ICF Core Set development methodology. If the aim is to review methodologies, the results should be organized more explicitly according to methodological components of Core Set development.

Major comment 9. Discussion and overinterpretation

The Discussion should be more closely aligned with the Results and should avoid overinterpretation. Several statements appear stronger than what is supported by the reported findings. For example, the authors state that "most CS developments adhered to the guidelines of Selb et al."; however, it is unclear whether adherence to Selb et al.'s methodological framework was systematically assessed. Similarly, the suggestion that the limited inclusion of Body Structures may be due to insufficient participation of medical specialists appears speculative unless supported by additional analysis.

The recommendations regarding global representation, multidisciplinary expert involvement, patient participation, validation, and implementation are important, but they should be clearly linked to the empirical findings of the review and phrased more cautiously where appropriate. Please revise the Discussion after reorganizing the Methods and Results, clearly distinguishing between findings directly supported by the review and the authors' broader recommendations for future ICF Core Set development.

Minor comments

Minor comment 1. References

Please carefully review the reference list for consistency. There appear to be discrepancies between the in-text citation numbering and the reference list, for example, reference

number 4 appears to be missing. In addition, journal titles such as Disability and Rehabilitation and reference formatting are not presented consistently. Please ensure that all references conform to the journal's citation style.

Minor comment 2. Paragraph structure in the Introduction

Consider starting a new paragraph before the sentence beginning with "This systematic scoping review aims...". Separating the study rationale from the study aim would improve readability and emphasize the transition from the literature review to the objectives of the present study.

Minor comment 3. Rationale for using a systematic scoping review

The authors state that a systematic scoping review was used because it allows mapping the breadth and depth of knowledge. While this rationale is appropriate, it would be helpful to explain more specifically why this approach was particularly suitable for reviewing methodologies used in ICF Core Set development.

Minor comment 4. Citation and numbered list formatting

Please ensure that in-text citations and numbered lists are formatted clearly and consistently according to BMJ Open's reference style. In several places, both citation numbers and numbered lists are presented in parentheses, which may reduce readability.

Minor comment 5. Conclusion

The Conclusion should be revised to more closely reflect the scope and limitations of the review. Some expressions appear too strong, such as "thoroughly analyzed," "it is essential," and "will improve." Given the methodological limitations and the primarily descriptive nature of the review, more cautious wording would be appropriate.

**Reviewer**	**2**
**Name**	**Natarajan, Jansirani**
**Affiliation**	**Department, College of Nursing, Sultan Qaboos University**
**Date**	**16-Jul-2026**
**COI**	**None**

Dear Authors,

Greetings.

It is a well-written scoping review manuscript.

Thank you

**Reviewer**	**3**

Name Ailey, Sarah H.

Affiliation Rush Univ, Community, Systems, and Mental Health Nursing

Date 24-Jul-2026

COI None

Below are my comments:

1. **Abstract and Introduction:** The abstract reflects the manuscript.

2. **Ethics:** This is a literature review and does not require IRB approval

3. **Appropriateness/adequacy of the methods.**
   The authors do not use consistent language. Most of the time they refer to "systematic scoping review" – for example in the abstract. On page 6 the term "scoping review" is used. At the bottom of page 32, the authors refer to "our systematic review." I note that Joanna Briggs has systematic criteria for scoping review, the term systematic scoping review is probably not needed and "scoping review" using Joanna Briggs guidelines would be enough.

   That said, the explanation of why a scoping review is needed is weak. The Joanna Briggs Institute does suggest describing a clear rationale for why a scoping review is needed. Common reasons include 1) identifying gaps in a research area that need to be addressed 2) identify types of study designs in a research area and/or types/range of outcomes 3) describing relevant contextual factors (Khalil et al., 2025). The introduction does indicate contextual factor and different types of study design. The issue of gaps is weak and the concluding paragraph on page 4 lines 25-32 is weak on summation of issues of why a scoping review. On page 6 under data extraction, linking to the phases described by Selb el al reads like a systematic review. Data extraction related to scoping review is not clearly described. Also, the paragraph as a whole does not make sense.

4. **Soundness of interpretations and relevance of discussion.**
   In the results, the authors do describe identify types of study designs/methodologies and range of outcomes in the development of the International Classification system. Though not described as such, there is description of some contextual factors. Gaps are described.

5. **Organization, clarity of writing, and adherence to format**
   Other than the issues raised above, the manuscript is clear and well-organized.

VERSION 1 - AUTHOR RESPONSE

Reviewer: 1

Overall comments

This manuscript addresses an important methodological topic: how ICF Core Sets have been developed across different health conditions and developmental phases. A review of this topic could provide valuable guidance for future ICF Core Set development, particularly regarding methodological transparency, patient and expert involvement, international representation, validation, and

implementation.
However, in its current form, the manuscript requires substantial revision. The main concerns relate to the clarity of the study rationale, reproducibility of the search strategy, specificity of the eligibility criteria, transparency of the study selection process, alignment between the stated objective and the data extraction framework, organization of the Results section, and consistency across the Abstract, Results, Figures, Tables, Appendices, and References. The Discussion and Conclusion should also be revised after the Methods and Results have been clarified, as several interpretations currently appear stronger than what is directly supported by the reported findings.

I therefore recommend major revision.

Thank you for your valuable feedback on our manuscript. We appreciate the time and effort you invested in reviewing our work and providing constructive comments. Your insights have helped us clarify and strengthen the presentation of our results. Below, we address your specific points in detail.

Major comment 1. Rationale and novelty of the review

The rationale for conducting this review could be strengthened. Although the authors cite previous reviews and state that existing guidelines are insufficiently specific, the manuscript does not clearly articulate the remaining knowledge gap. It would be helpful to explain more explicitly why previous reviews are insufficient, what methodological questions remain unanswered, and how the present review uniquely addresses these gaps. This would strengthen the novelty and significance of the study.

Thank you for this important comment. We agree that the rationale and novelty of the review were not sufficiently articulated in the original manuscript. We have revised the final section of the Introduction to more clearly describe the remaining knowledge gap. Specifically, we now explain that previous reviews were either outdated or focused on a single phase of ICF Core Set development, leaving unanswered questions regarding methodological variation across the entire development process. We further clarify that no previous review has systematically examined how researchers operationalised the broad methodological recommendations for ICF Core Set development, including stakeholder involvement, geographical representation, and validation approaches.

We changed the last part of the introduction to the following:

> *Given the substantial increase in the number of ICF CS developed over the past decade, a contemporary synthesis of development methodologies is needed. Such an overview can identify methodological trends, areas of inconsistency, and potential gaps in current practice, thereby providing guidance for future CS development initiatives. Therefore, this systematic scoping review aimed to examine the methods and techniques used in the development of ICF CS between 2001 and 2024 across all developmental phases. By mapping methodological variation, stakeholder involvement, geographical representation, and validation approaches, this review seeks to provide practical recommendations for the future development of robust, internationally applicable ICF CS.*

Major comment 2. Search strategy and reliance on PubMed

Although the authors acknowledge the limitation of relying primarily on PubMed, the justification provided mainly focuses on whether all existing ICF Core Sets were identified. However, the aim of this review is not merely to identify ICF Core Sets, but to review, classify, and analyze the methodologies

and techniques used in their development. Therefore, the key issue is whether all relevant publications describing the development methodology of each Core Set were sufficiently identified.

The authors should clarify this distinction and discuss more explicitly how the PubMed-based search strategy may have affected the completeness of the methodological analysis. The limitations section should also be revised to distinguish between identifying existing Core Sets and identifying all relevant publications needed to analyze their development methodologies.

We agree that identifying existing ICF Core Sets and identifying all methodological publications related to their development are two distinct issues. In the revised manuscript, we clarified this distinction in both the Methods and Limitations sections.

Methods section:

> *As ICF Core Sets are centrally documented and publicly accessible via the official ICF Research Branch website, we expected that the identification of existing CSs would be relatively complete when using PubMed combined with reference screening and author contact. However, the objective of this review extended beyond identifying CSs and included examining the methodological studies underpinning their development. To improve methodological coverage, reference lists of included studies were screened and additional publications were sought through direct contact with authors when necessary.*

Limitations:

> *Our systematic review relied primarily on the PubMed database and therefore may not have captured all methodological publications related to ICF Core Set development. While the official ICF Core Sets platform (https://www.icf-core-sets.org/) enabled us to verify the inclusion of all existing CSs, this does not necessarily guarantee that all publications describing preparatory studies, consensus procedures, validation activities, or implementation work associated with those CSs were identified. To mitigate this risk, we supplemented the database search through reference screening and direct communication with authors. Nevertheless, methodological publications indexed exclusively in databases such as CINAHL, PsycINFO, Embase, or Scopus may have been missed. Consequently, some methodological variations may not have been fully captured in this review.*

Major comment 3. Full search strategy should be reported

The authors state that relevant publications were identified in PubMed using standardized MeSH terms combined with Boolean expressions. However, the specific MeSH terms, free-text terms, Boolean operators, and full PubMed search strategy are not reported. This substantially limits the reproducibility and transparency of the review, particularly because PubMed was used as the primary database.

Please provide the complete PubMed search strategy, including all search terms, Boolean operators, limits, and date of search, preferably in the Methods section or as supplementary material.

We included the reference to appendix 2 in the Method section, where the search strategy is now fully provided.

Major comment 4. Eligibility criteria and study selection process

The eligibility criteria and study selection process require substantial clarification. The criterion of "publications reporting on the development of Core Sets" is broad, and it is unclear which types of publications were eligible, such as protocol papers, preparatory studies, consensus conference reports, validation studies, implementation studies, related methodological papers, or studies on ICF-based assessment sets and other ICF-derived tools. This lack of specificity limits the transparency and reproducibility of the study selection process.

Moreover, Figure 2 is internally inconsistent: 507 screened records minus 341 excluded records would leave 166 records, whereas 170 reports are shown as assessed for eligibility. The diagram also does not report exclusion reasons, duplicate removal, or whether any reports were excluded after full-text assessment. Please clarify the eligibility criteria and revise the PRISMA flow diagram with consistent numbers and clear exclusion reasons.

In the revised manuscript, we clarified the inclusion and exclusion criteria by explicitly specifying the types of publications that were eligible for inclusion, including preparatory studies (e.g., systematic reviews, patient perspective studies, and expert surveys), protocol or invitation papers, consensus conference reports, validation studies, and implementation studies related to ICF Core Set development.

> *Because the ICF was officially launched in 2001, only publications reporting on any phase of the development of ICF CS that were published between January 2001 and July 2024 were included. Eligible publications included preparatory studies (e.g., systematic reviews, patient perspective studies, and expert surveys), protocol or invitation papers, consensus conference reports, validation studies, and implementation studies. Additionally, publications had to be written in English and the research had to be performed on human subjects. Publications were excluded if they were not related to the development of CS.*

In addition, we revised the PRISMA flow diagram to improve transparency and consistency. The updated flowchart now provides more detailed information on the study selection process, including clearer reporting of exclusions and exclusion categories, allowing readers to better understand how studies were identified, screened, assessed for eligibility, and included in the review. These revisions improve the transparency, reproducibility, and overall clarity of the study selection process.

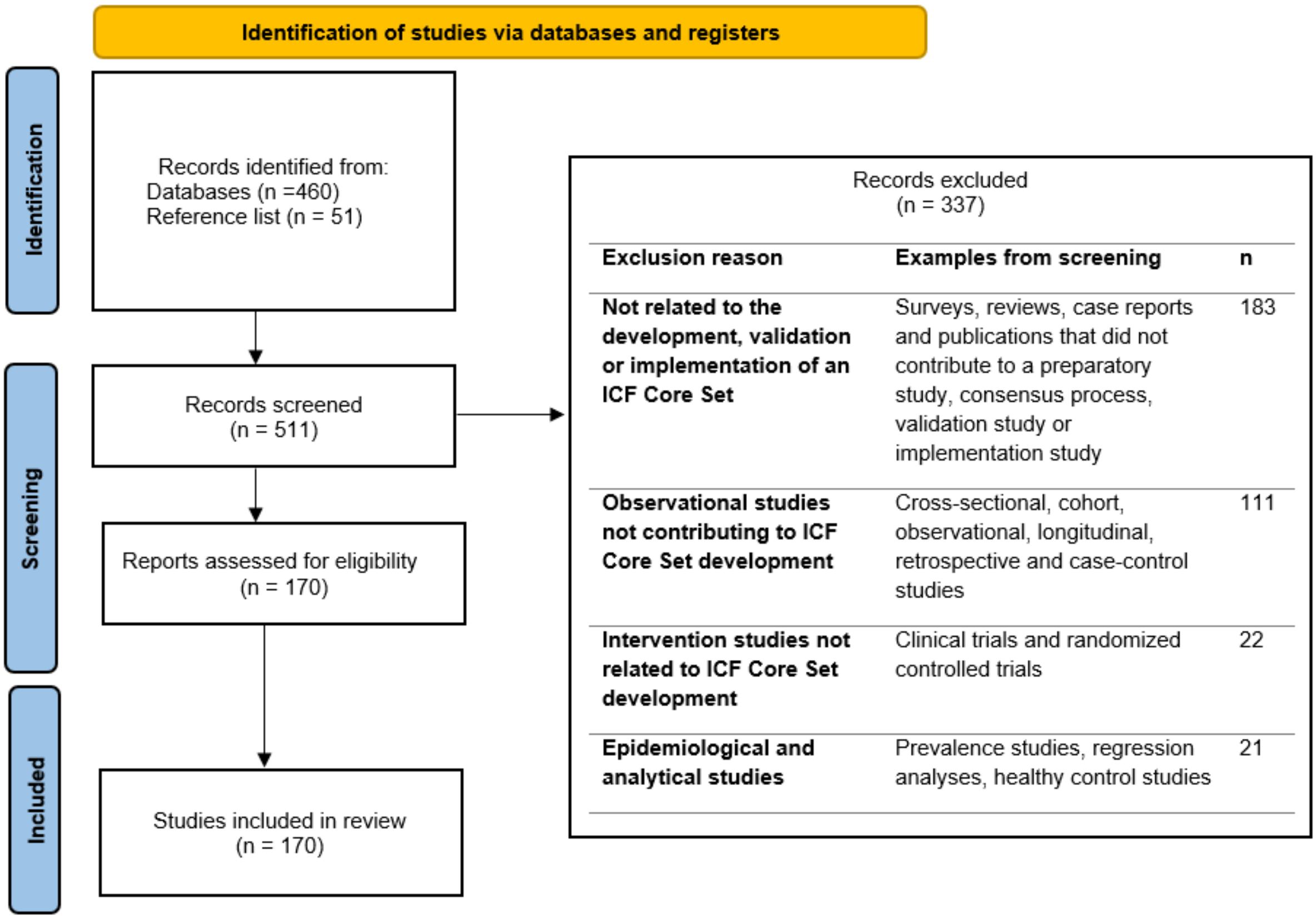

Major comment 5. Data extraction and analytical framework

The data extraction and analysis methods require further clarification. The authors state that the identified publications were linked to the developmental phases outlined by Selb et al.; however, it is unclear how this linkage was performed, who conducted the classification, whether multiple reviewers were involved, how disagreements were resolved, and how studies covering more than one developmental phase were handled.

In addition, the criteria used to assess the practical use and generalizability of developed Core Sets are not clearly described. Given that the aim of this review is to examine methodologies used in ICF Core Set development, the data extraction framework should be described in more detail and should be more explicitly aligned with the methodological components of Core Set development.

We agree that the description of the data extraction and analytical framework required additional detail. In the revised manuscript, we have clarified how publications were classified according to the developmental phases proposed by Selb et al., including the criteria used for assigning studies

> *The identified publications, were classified according to the developmental framework by Selb et al. (3) and looked at reports on possibilities to use developed CS in practice and their generalizability to other settings and populations. Classification was based on the primary objective and methodology reported in each publication. Studies describing systematic reviews, patient perspective studies, expert surveys and protocol/discussion papers were assigned to the preparatory phase. Publications reporting on international*

consensus conferences were assigned to Phase I. Validation studies, implementation studies and studies evaluating the applicability of an ICF CS in specific professional groups, settings or countries were assigned to Phase II. When publications addressed more than one developmental phase, they were linked to all relevant phases and were considered in each corresponding section of the analysis.

Data extraction was performed using a structured extraction framework aligned with the methodological components of ICF CS development. Extracted data included study type, developmental phase, study methodology, sample size, participant characteristics, professional disciplines involved, country and WHO region, consensus procedures, validation activities, implementation activities, publication year and journal.

To provide insight into the practical applicability and generalizability of developed Core Sets, information was extracted regarding the populations involved, professional groups represented, geographical distribution of participants, and the extent to which studies included stakeholders from multiple WHO regions. An overview of all included and linked studies can be found in appendix 1.

Major comment 6. Organization and presentation of the Results section

The Results section requires substantial reorganization and rewriting. At present, the presentation of results is difficult to follow, and the main findings are not clearly prioritized. The text often provides lengthy descriptive details, but it is not always clear how these findings relate to the study objective or to the analytical framework described in the Methods. In particular, the sections on expert perspectives, Phase I, and Phase II mix descriptions of study designs, participant characteristics, consensus methods, validation procedures, and implementation issues without a clear structure.

Please reorganize the Results section so that it is explicitly aligned with the study aim and methods. The main methodological findings should be presented in a logical order, preferably based on the predefined ICF Core Set development phases and/or clearly defined methodological categories. Figures and tables should serve as the primary means of presenting detailed data, while the main text should highlight and interpret the key findings rather than repeat or loosely summarize the tables. This restructuring would substantially improve the clarity, readability, and methodological contribution of the review.
For example, Figure 3 would be easier to interpret if exact numbers were shown and if the correspondence between categories and developmental phases was clearer.

We agree that the original Results section contained a substantial amount of descriptive information, which reduced the visibility of the main methodological findings. In the revised manuscript, we have thoroughly reorganized the Results section.

And we added a new section entitled **“**methodological findings**”**, which provides a concise synthesis of the main patterns identified across all developmental phases.

New structure:

1. Study selection and characteristics

2. Methodologies used in the preparatory phase

2.1 Study protocol/discussion papers

2.2 Literature reviews

2.3 Patient perspective studies

2.4 Expert surveys

3. Methodologies used in Phase I

4. Methodologies used in Phase II

5. Methodological findings

Major comment 7. Consistency of units of analysis, figures, and tables

The Results section would benefit from clearer organization and greater consistency in the unit of analysis. The current section mixes results reported at the level of publications, studies, and Core Sets, making it difficult to interpret the findings. For example, the Abstract reports 36 Core Sets, whereas the Results section states that 34 Core Sets were addressed. In addition, it is unclear whether the percentages by health condition are based on publications or Core Sets.

The references to tables also appear inconsistent. For example, Table 1 seems to present the number of ICF categories in comprehensive and brief Core Sets, rather than the distribution of health conditions, while Table 2 appears to describe characteristics of systematic reviews rather than the distribution of ICF components. Please clarify the unit of analysis throughout the Results section, ensure consistency between the Abstract, Results, Tables, Figures, and Appendices, and reorganize the results according to the methodological focus of the review.

In the revised manuscript, we corrected discrepancies

Major comment 8. Alignment between Selb et al.'s framework and the presented classifications

Although the authors state that included publications were linked to the developmental phases outlined by Selb et al., the variables presented in the tables do not always appear to be directly derived from, or clearly mapped onto, Selb et al.'s methodological framework for ICF Core Set development. For example, Table 2 mainly summarizes general characteristics of systematic reviews, such as inclusion/exclusion criteria, databases, and sample size.

Please clarify how the table variables were selected and how they relate to the established ICF Core Set development methodology. If the aim is to review methodologies, the results should be organized more explicitly according to methodological components of Core Set development.

In the revised manuscript, we further aligned the presentation of results with the methodological phases of ICF Core Set development described by Selb et al. Publications are now classified according to the preparatory phase, consensus phase, and validation/implementation phase. See also major comment 6.

In response to this suggestion, Figure 3 was revised to display the exact numbers for each category. We believe this improves interpretability and allows readers to more easily compare the distribution of publications across the developmental phases of ICF Core Set development.

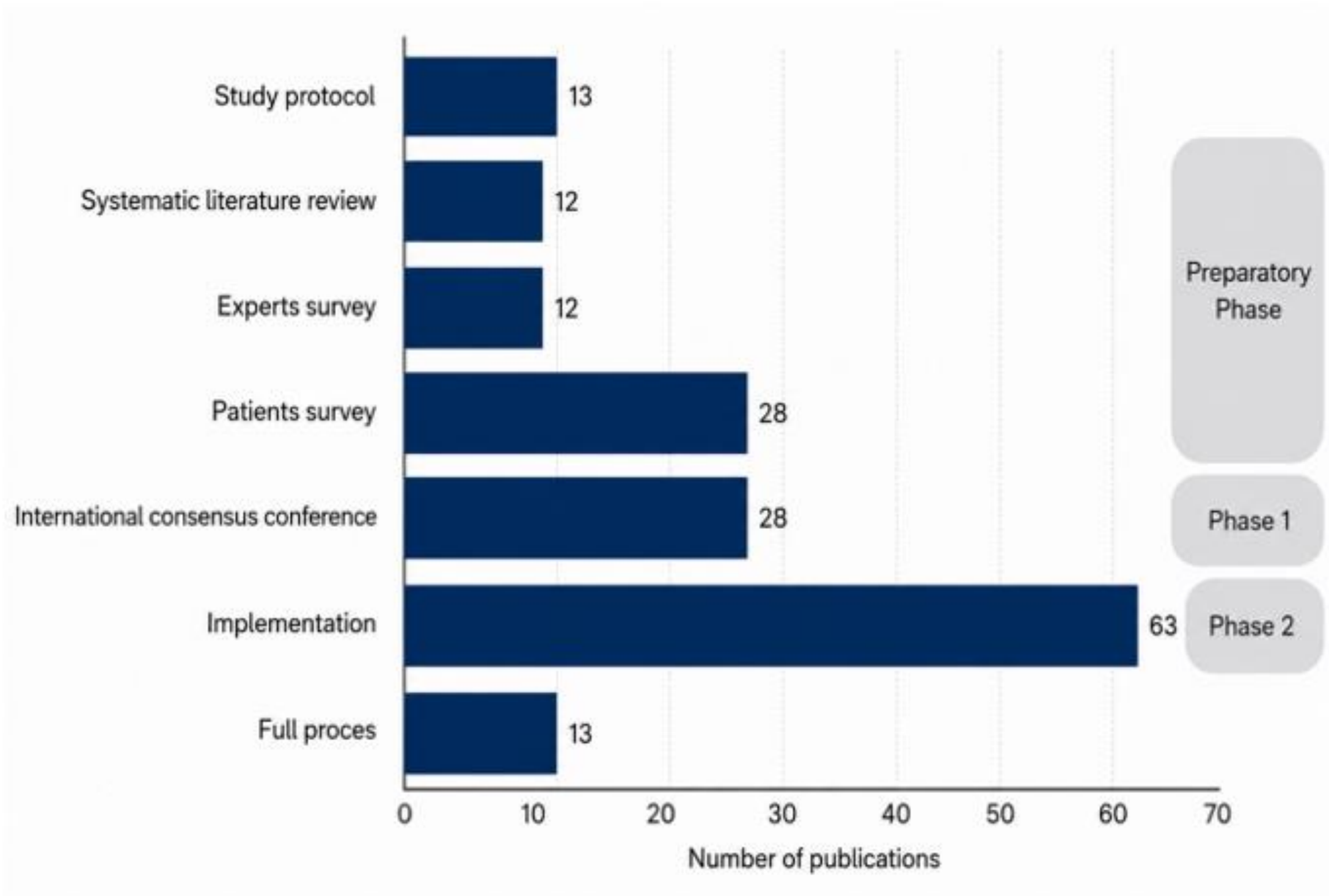

Major comment 9. Discussion and overinterpretation

The Discussion should be more closely aligned with the Results and should avoid overinterpretation. Several statements appear stronger than what is supported by the reported findings. For example, the authors state that "most CS developments adhered to the guidelines of Selb et al."; however, it is unclear whether adherence to Selb et al.'s methodological framework was systematically assessed. Similarly, the suggestion that the limited inclusion of Body Structures may be due to insufficient participation of medical specialists appears speculative unless supported by additional analysis.

The recommendations regarding global representation, multidisciplinary expert involvement, patient participation, validation, and implementation are important, but they should be clearly linked to the empirical findings of the review and phrased more cautiously where appropriate. Please revise the Discussion after reorganizing the Methods and Results, clearly distinguishing between findings directly supported by the review and the authors' broader recommendations for future ICF Core Set development.

We agree that several statements in the original Discussion were stronger than warranted by the findings of the review. In the revised manuscript, we carefully reviewed the Discussion section and distinguished more clearly between findings directly supported by the data and recommendations for future research. Speculative interpretations were either removed or reformulated more cautiously.

Minor comments

Minor comment 1. References

Please carefully review the reference list for consistency. There appear to be discrepancies between the in-text citation numbering and the reference list, for example, reference number 4 appears to be missing. In addition, journal titles such as Disability and Rehabilitation and reference formatting are not presented consistently. Please ensure that all references conform to the journal's citation style.

The reference table has been updated to improve clarity and consistency.

Minor comment 2. Paragraph structure in the Introduction

Consider starting a new paragraph before the sentence beginning with "This systematic scoping review aims...". Separating the study rationale from the study aim would improve readability and emphasize the transition from the literature review to the objectives of the present study.

We agree, in the revised manuscript, we have restructured the Introduction by introducing a new paragraph before the statement describing the aim of the review.

Minor comment 3. Rationale for using a systematic scoping review

The authors state that a systematic scoping review was used because it allows mapping the breadth and depth of knowledge. While this rationale is appropriate, it would be helpful to explain more specifically why this approach was particularly suitable for reviewing methodologies used in ICF Core Set development.

We have expanded the rationale for using a systematic scoping review in the Methods section:

> *The method of a systematic scoping review was used because it provided the opportunity to map the breadth and depth of knowledge in the development of a CS and provided opportunities to identify gaps in the available knowledge. This approach was considered particularly appropriate because the objective of the review was to examine and compare the wide range of methodologies used across the preparatory, consensus, and validation phases of ICF CS development rather than to evaluate intervention effectiveness or synthesize outcome data.*

Minor comment 4. Citation and numbered list formatting

Please ensure that in-text citations and numbered lists are formatted clearly and consistently according to BMJ Open's reference style. In several places, both citation numbers and numbered lists are presented in parentheses, which may reduce readability.

We carefully reviewed the manuscript and, where appropriate, revised the formatting of in-text citations and numbered lists to improve readability and ensure greater consistency throughout the manuscript.

Minor comment 5. Conclusion

The Conclusion should be revised to more closely reflect the scope and limitations of the review. Some expressions appear too strong, such as "thoroughly analyzed," "it is essential," and "will improve." Given the methodological limitations and the primarily descriptive nature of the review, more cautious wording would be appropriate.

We have revised the Conclusion to better reflect the scope and limitations of this review. We softened several statements and ensured that the conclusions and recommendations are more closely aligned with the findings presented in the Results and Discussion sections.

> *We conducted a systematic scoping review to investigate the past developmental methodologies of ICF CS. Our study contributes to understanding the different methodologies used. We thoroughly described and synthesized the different phases of the CS development process, the level of expert and patient involvement, and the observed variation across different initiatives. Notably, the methodology of the included studies varied significantly, and there was limited representation of studies from outside Europe. To enhance the practical relevance of future CS, it is essential to carefully consider and integrate the perspectives of potential users, including patients and experts from all WHO regions. Based on the findings of this review, future ICF CS developments may benefit from three key considerations. First, broader patient involvement across developmental phases may improve relevance and acceptability. Second, more balanced representation across WHO regions may strengthen international applicability. Third, greater consistency in validation and implementation methodologies may facilitate comparison across CSs and support wider uptake in clinical practice. These recommendations should be interpreted as potential directions for future development rather than conclusions directly demonstrated by the included studies.*

Reviewer: 2

Dr. Jansirani Natarajan, Department, College of Nursing, Sultan Qaboos University
Comments to the Author:
Dear Authors,
Greetings.
It is a well-written scoping review manuscript.
Thank you

We thank the reviewer for the positive evaluation of our manuscript and for taking the time to review our work.

Reviewer: 3

Dr. Sarah H. Ailey, Rush Univ
Comments to the Author:
Re: bmjopen -2026-123955
7/24/26

Thank you for the opportunity to review this manuscript. Improvements in early diagnosis of autism an the use of databases in early diagnosis is an important topic.

Thank you for this positive assessment. We appreciate the reviewer’s observation that the review identifies methodological approaches, study designs, outcomes, contextual factors, and knowledge gaps related to ICF Core Set development. In the revised manuscript, we further strengthened the

rationale for conducting a scoping review and clarified the data extraction and analysis procedures to better demonstrate how methodological variation, contextual factors, and research gaps were mapped across the different phases of ICF Core Set development.

(We believe the comment about autism was not meant to be part of the feedback on our manuscript).

Below are my comments:

1. Abstract and Introduction: The abstract reflects the manuscript.

Thank you.

2. Ethics: This is a literature review and does not require IRB approval

We agree.

3. Appropriateness/adequacy of the methods.

The authors do not use consistent language. Most of the time they refer to "systematic scoping review" – for example in the abstract. On page 6 the term "scoping review" is used. At the bottom of page 32, the authors refer to "our systematic review." I note that Joanna Briggs has systematic criteria for scoping review, the term systematic scoping review is probably not needed and "scoping review" using Joanna Briggs guidelines would be enough.

That said, the explanation of why a scoping review is needed is weak. The Joanna Briggs Institute does suggest describing a clear rationale for why a scoping review is needed. Common reasons include 1) identifying gaps in a research area that need to be addressed 2) identify types of study designs in a research area and/or types/range of outcomes 3) describing relevant contextual factors (Khalil et al., 2025). The introduction does indicate contextual factor and different types of study design. The issue of gaps is weak and the concluding paragraph on page 4 lines 25-32 is weak on summation of issues of why a scoping review. On page 6 under data extraction, linking to the phases described by Selb el al reads like a systematic review. Data extraction related to scoping review is not clearly described. Also, the paragraph as a whole does not make sense.

We agree that terminology should be used consistently throughout the manuscript. In the revised version, we carefully reviewed the manuscript and harmonized the terminology. We chose to retain the term *systematic scoping review* throughout the manuscript, as the review was conducted using a systematic and transparent methodology in accordance with PRISMA-ScR and Joanna Briggs Institute guidance. We believe this terminology accurately reflects both the exploratory nature of the review and the rigorous methodological approach that was applied.

We also strengthened the rationale for conducting a systematic scoping review:

> *The method of a systematic scoping review was used because it provided the opportunity to map the breadth and depth of knowledge in the development of any CS and provided opportunities to identify gaps in the available knowledge. This approach was considered particularly appropriate because the objective of the review was to examine and compare the wide range of methodologies used across the preparatory, consensus, and validation phases of ICF CS development rather than to evaluate intervention effectiveness or synthesize outcome data.*

Furthermore, we revised the Data Extraction and Analysis section to clarify how publications were mapped to the developmental phases proposed by Selb et al. and which methodological variables were extracted. This clarification better reflects the exploratory and mapping purpose of a scoping review.

4. Soundness of interpretations and relevance of discussion.
In the results, the authors do describe identify types of study designs/methodologies and range of outcomes in the development of the International Classification system. Though not described as such, there is description of some contextual factors. Gaps are described.

Thank you for this positive observation.

5. Organization, clarity of writing, and adherence to format
Other than the issues raised above, the manuscript is clear and well-organized.

Thank you for your positive appraisal of our work.

VERSION 2 - REVIEW

**Reviewer**	**3**
**Name**	**Ailey, Sarah H.**
**Affiliation**	**Rush Univ, Community, Systems, and Mental Health Nursing**
**Date**	**31-Aug-2026**
**COI**	

Re: bmjopen -2026-123955
8/31/26

Thank you for the opportunity to re-review this manuscript. The authors have responded thoughtfully to the previous comments and have improved the manuscript. This systematic scoping review addresses an important topic and provides a useful overview of methodologies used in the development of ICF Core Sets. The manuscript is generally well organized and, following revision, provides a clearer rationale for the use of a scoping review. My remaining comments are provided below.

1. Abstract and Introduction

The rationale for conducting a scoping review is now more clearly articulated.

The statement "there is a need to shortlist these categories for specific health conditions to describe the functioning and disabilities of particular groups of people" would benefit from additional justification or citation. This may be a commonly accepted premise underlying ICF Core Set development; however, the authors do not provide a citation or justification for this

statement. In addition, it is not a conclusion that emerges directly from the findings of the present review.

The developmental framework proposed by Selb et al. is central to the organization and interpretation of this review. Explanation of this framework in the introduction would help orient readers and provide a clearer rationale for the subsequent analyses. As well, the authors mention the ICF linking rules proposed by Cieza et al. first in the Results section without explanation of these rules. The rules proposed by Cieza et al. also appear to be central to the organization and interpretation of this review. Because the review relies heavily on concepts in the Selb framework and the ICF linking rules, these methodological foundations should be introduced and briefly explained in the Introduction. The Cieza et al linking rules should be mentioned in the abstract.

2. Ethics

As this study is a literature review and did not involve human participants, ethics approval or IRB review was not required. The statement "Not applicable" is sufficient.

3. Appropriateness and Adequacy of the Methods

The methodology is generally appropriate for the stated aims. The use of the Selb framework to organize studies across developmental phases provides a logical structure for the review.

4. Soundness of Interpretations and Relevance of the Discussion

The Results provide a useful description of the study designs and methodologies used in ICF Core Set development. Although not explicitly framed this way, the review also identifies important contextual factors influencing development processes. The manuscript highlights several methodological gaps and provides practical suggestions for future Core Set development.

One point that should be reconsidered is the statement that patients were "always involved" in development. Based on the findings presented, patient involvement appears common and increasingly emphasized, particularly during preparatory phases, but not universal across all developmental activities. It would be more accurate to state that patients participated in many or most preparatory studies rather than implying universal involvement.

The discussion appropriately notes the evolution of ICF Core Set development methodologies and may help inform efforts toward greater methodological consistency across future developments.

5. Organization, Clarity of Writing, and Adherence to Format

Other than the issues noted above, the manuscript is clearly written, logically organized, and generally adheres to journal expectations. The tables are particularly useful in summarizing methodological characteristics across studies.

Overall Assessment

This manuscript provides a valuable overview of ICF Core Set development methodologies and offers practical recommendations for future work. The authors have addressed many of the concerns raised during the initial review. Addressing the remaining points above would further strengthen the clarity and methodological grounding of the paper.

VERSION 2 - AUTHOR RESPONSE

Response to reviewers

Thank you for the opportunity to re-review this manuscript. The authors have responded thoughtfully to the previous comments and have improved the manuscript. This systematic scoping review addresses an important topic and provides a useful overview of methodologies used in the development of ICF Core Sets. The manuscript is generally well organized and, following revision, provides a clearer rationale for the use of a scoping review. My remaining comments are provided below.

We would like to thank the reviewer for the careful re-evaluation of our manuscript and for the constructive and encouraging feedback. We appreciate the recognition that the manuscript has improved following revision and that the rationale for undertaking a scoping review is now more clearly articulated. We are pleased that the reviewer considers this review a valuable contribution to the literature on ICF Core Set development methodologies.

In response to the remaining comments, we have made several additional revisions to further strengthen the manuscript. Below, we provide a detailed point-by-point response to each comment and indicate the corresponding changes made in the revised manuscript.

Abstract and Introduction

The rationale for conducting a scoping review is now more clearly articulated. The statement "there is a need to shortlist these categories for specific health conditions to describe the functioning and disabilities of particular groups of people" would benefit from additional justification or citation. This may be a commonly accepted premise underlying ICF Core Set development; however, the authors do not provide a citation or justification for this statement. In addition, it is not a conclusion that emerges directly from the findings of the present review.

The developmental framework proposed by Selb et al. is central to the organization and interpretation of this review. Explanation of this framework in the introduction would help orient readers and provide a clearer rationale for the subsequent analyses. As well, the authors mention the ICF linking rules proposed by Cieza et al. first in the Results section without explanation of these rules. The rules proposed by Cieza et al. also appear to be central to the organization and interpretation of this review. Because the review relies heavily on concepts in the Selb framework and the ICF linking rules, these

methodological foundations should be introduced and briefly explained in the Introduction. The Cieza et al linking rules should be mentioned in the abstract.

We thank the reviewer for this valuable suggestion. We have strengthened the Introduction by providing a clearer rationale for the development of ICF Core Sets, including an additional justification and citation for the need to reduce the number of ICF categories. We have also expanded the description of the developmental framework proposed by Selb et al. and introduced the ICF linking rules developed by Cieza et al. in the Introduction.

> However, due to the extensive nature of the ICF, which consists of 1,424 categories, To enhance feasibility and clinical usability, there is a need to shortlist these categories for specific health conditions to describe the functioning and disabilities of particular groups of people condition-specific ICF Core Sets were developed (3). These Core Sets aims to identify the most relevant ICF categories needed to describe functioning, disability and contextual factors for specific health conditions while retaining the biopsychosocial perspective of the ICF framework.. There are comprehensive Core Sets (CS), consisting of all ICF categories relevant for a specific condition, and brief CS, which are more compressed versions of the comprehensive CS (3).
>
> The development of an ICF CS is commonly guided by the framework described by Selb et al. (3), which provides methodological recommendations for the creation of condition-specific CSs. This framework consists of three interrelated phases. First, a preparatory phase gathers evidence from different perspectives, including the research, patient, and expert perspectives, through systematic reviews, qualitative studies and expert surveys. Second, an international consensus process is used to determine which ICF categories should be included in the comprehensive and brief CSs. Third, the resulting CSs undergo validation and implementation studies to evaluate their relevance, applicability and feasibility across settings and populations. Because this framework has become the dominant approach for ICF Core Set development, it was used as the organizing structure for the present review. (Figure 1)A key methodological component of many ICF CS development studies is the use of the ICF linking rules developed by Cieza et al. (4,5). These rules provide a standardized procedure for linking concepts identified in research evidence, patient interviews, questionnaires, and expert surveys to the most appropriate ICF categories. The linking rules support consistency and transparency across preparatory studies and have become a cornerstone of ICF CS methodology.

Soundness of Interpretations and Relevance of the Discussion

The Results provide a useful description of the study designs and methodologies used in ICF Core Set development. Although not explicitly framed this way, the review also identifies important contextual factors influencing development processes. The manuscript highlights several methodological gaps and provides practical suggestions for future Core Set development.

One point that should be reconsidered is the statement that patients were "always involved" in development. Based on the findings presented, patient involvement appears common and increasingly emphasized, particularly during preparatory phases, but not universal across all developmental activities. It would be more accurate to state that patients participated in many or most preparatory studies rather than implying universal involvement.

The discussion appropriately notes the evolution of ICF Core Set development methodologies and may help inform efforts toward greater methodological consistency across future developments.

We thank the reviewer for this observation. We agree that the wording "patients were always involved" was too absolute and not fully supported by the data presented. We have revised the text throughout the manuscript

Page 17 Line 6:

> Patients were frequently involved during the preparatory phase, primarily through focus groups, interviews, and questionnaires.
