# Supplementary material for: Methodologies used in the development of the international classification of functioning, disability and health core sets: a systematic scoping review
Source: BMJ Open. 2026 Oct 5;16(10):e123955. doi: 10.1136/bmjopen-2026-123955 (PMC13646977; doi:10.1136/bmjopen-2026-123955)
Supplement: Supplementary file 2 [file bmjopen-16-10-s001.docx]

**Appendix 1: Overview included CS**

| **Core set name** | **Invitation paper** | **Systematic review** | **Patient survey** | **Expert survey** | **Consensus meeting** | **Implementation** |  |
| --- | --- | --- | --- | --- | --- | --- | --- |
| **Musculoskeletal Conditions** | | | | | | | |
| **Chronic pain** |  |  |  |  | Cieza A, Stucki G, Weigl M, Kullmann L, Stoll T, Kamen L, et al. ICF Core Sets for chronic widespread pain. Journal of Rehabilitation Medicine, Supplement. 2004. p. 63–8. |  |  |
| **Ankylosing spondylitis** | Boonen A, Rasker JJ, Stucki G. The international classification for functioning, disability and health: A challenge and a need for rheumatology. Vol. 26, Clinical Rheumatology. 2007. p. 1803–8. |  | Boonen A, Van Berkel M, Cieza A, Stucki G, Van Der Heijde D. Which aspects of functioning are relevant for patients with ankylosing spondylitis: Results of focus group interviews. Journal of Rheumatology. 2009 Nov;36(11):2501–11.  Van Echteld I, Cieza A, Boonen A, Stucki G, Zochling J, Braun J, et al. Identification of the Most Common Problems by Patients with Ankylosing Spondylitis Using the International Classification of Functioning, Disability and Health. J Rheumatol [Internet]. 2006;33(12):2475–83. | Boonen A, van Berkel M, Kirchberger I, Cieza A, Stucki G, van der Heijde D. Aspects relevant for functioning in patients with ankylosing spondylitis according to the health professionals: A Delphi study with the ICF as reference. Rheumatology. 2009;48(8):997–1002. |  |  |  |
| **Osteoporosis** |  |  |  |  | Cieza A, Schwarzkopf SR, Sigl T, Stucki G, Melvin J, Stoll T, et al. ICF Core Sets for osteoporosis. Vol. Supplement, Journal of Rehabilitation Medicine. 2004. p. 81–6. | Koehler B, Kirchberger I, Glaessel A, Kool J, Stucki G, Cieza A. Validation of the International Classification of Functioning, Disability and Health Comprehensive Core Set for Osteoporosis: The perspective of physical therapists. Vol. 34, Journal of Geriatric Physical Therapy. Lippincott Williams and Wilkins; 2011. p. 117–30. |  |
| **Low back pain** |  |  | Røe C, Sveen U, Bautz-Holter E. Retaining the patient perspective in the International Classification of Functioning, Disability and Health Core Set for low back pain. Vol. 2, Patient Preference and Adherence. 2008. |  | Cieza A, Stucki G, Weigl M, Disler P, Jäckel W, van der Linden S, et al. ICF Core Sets for low back pain. Journal of Rehabilitation Medicine, Supplement. 2004. p. 69–74. | Glocker C, Kirchberger I, Gläßel A, Fincziczki A, Stucki G, Cieza A. Content validity of the comprehensive international classification of functioning, disability and health (ICF) core set for low back pain from the perspective of physicians: A Delphi survey. Vol. 9, Chronic Illness. 2013. p. 57–72.  Kirschneck M, Kirchberger I, Amann E, Cieza A. Validation of the comprehensive ICF core set for low back pain: The perspective of physical therapists. Man Ther. 2011 Aug;16(4):364–72.  Mullis R, Barber J, Lewis M, Hay E. ICF core sets for low back pain: Do they include what matters to patients? J Rehabil Med. 2007 May;39(5):353–7. |  |
| **osteoarthritis** |  |  | Stamm TA, Cieza A, Coenen M, Machold KP, Nell VPK, Smolen JS, et al. Validating the International Classification of Functioning, Disability and Health Comprehensive Core Set for Rheumatoid Arthritis from the patient perspective: A qualitative study. Arthritis Care Res (Hoboken). 2005 Jun 15;53(3):431–9. |  | Grill E, Zochling J, Stucki G, Mittrach R, Scheuringer M, Liman W, et al. International Classification of Functioning, Disability and Health (ICF) Core Set for patients with acute arthritis. Vol. 25, Clinical and Experimental Rheumatology. 2007.  Dreinhöfer K, Stucki G, Ewert T, Huber E, Ebenbichler G, Gutenbrunner C, et al. ICF Core Sets for osteoarthritis. Journal of Rehabilitation Medicine, Supplement. 2004. p. 75–80. | Uhlig T, Lillemo S, Moe RH, Stamm T, Cieza A, Boonen A, et al. Reliability of the ICF Core Set for rheumatoid arthritis. Ann Rheum Dis. 2007 Aug;66(8):1078–84.  Oberhauser C, Escorpizo R, Boonen A, Stucki G, Cieza A. Statistical validation of the brief international classification of functioning, disability and health core set for osteoarthritis based on a large international sample of patients with osteoarthritis. Arthritis Care Res (Hoboken). 2013 Feb;65(2):177–86.  Stucki G, Cieza A. The International Classification of Functioning, Disability and Health (ICF) core sets for rheumatoid arthritis: A way to specify functioning. In: Annals of the Rheumatic Diseases. 2004.  Gebhardt C, Kirchberger I, Stucki G, Cieza A. Validation of the comprehensive icf core set for rheumatoid arthritis: The perspective of physicians. J Rehabil Med. 2010 Sep;42(8):780–8.  Kirchberger I, Cieza A, Stucki G. Validation of the Comprehensive ICF Core Set for rheumatoid arthritis: The perspective of psychologists. Psychol Health. 2008 Aug;23(6):639–59.  Kirchberger I, Glaessel A, Stucki G, Cieza A. Validation of the Comprehensive International Classification of Functioning, Disability and Health Core Set for Rheumatoid Arthritis: The Perspective of Physical Therapists Background and Purpose [Internet]. 2007.  Uhlig T, Moe R, Reinsberg S, Kvien TK, Cieza A, Stucki G. Responsiveness of the International Classification of Functioning, Disability and Health (ICF) Core Set for rheumatoid arthritis. Ann Rheum Dis. 2009 Jun;68(6):879–84.  Xie F, Thumboo J, Fong KY, Lo NN, Yeo SJ, Yang KY, et al. Are they relevant? A critical evaluation of the international classification of functioning, disability, and health core sets for osteoarthritis from the perspective of patients with knee osteoarthritis in Singapore. Ann Rheum Dis. 2006 Aug;65(8):1067–73. |  |
| **Neurologiscal conditions** | | | | | | | |
| **Cerebral palsy** |  | Benner JL, Noten S, Limsakul C, Van Der Slot WMA, Stam HJ, Selb M, et al. Outcomes in adults with cerebral palsy: systematic review using the International Classification of Functioning, Disability and Health. Vol. 61, Developmental Medicine and Child Neurology. Blackwell Publishing Ltd; 2019. p. 1153–61. | Noten S, Troenosemito LAA, Limsakul C, Selb M, de Groot V, Konijnenbelt M, et al. Development of an ICF Core Set for adults with cerebral palsy: capturing their perspective on functioning. Dev Med Child Neurol. 2021 Jul 1;63(7):846–52.  Schiariti V, Sauve K, Klassen AF, O’Donnell M, Cieza A, Mâsse LC. “He does not see himself as being different”: The perspectives of children and caregivers on relevant areas of functioning in cerebral palsy. Dev Med Child Neurol. 2014;56(9):853–61. | Schiariti V, Mâsse LC, Cieza A, Klassen AF, Sauve K, Armstrong R, et al. Toward the development of the international classification of functioning core sets for children with cerebral palsy: A global expert survey. J Child Neurol. 2014;29(5):582–91.  Limsakul C, Noten S, Selb M, Stam HJ, Van Der Slot WMA, Roebroeck ME. Developing an ICF Core Set for adults with cerebral palsy: A Global expert survey of relevant functions and contextual factors. Vol. 52, Journal of Rehabilitation Medicine. Foundation for Rehabilitation Information; 2020. | Schiariti V, Selb M, Cieza A, O’Donnell M. International Classification of Functioning, Disability and Health Core Sets for children and youth with cerebral palsy: A consensus meeting. Dev Med Child Neurol. 2015 Feb 1;57(2):149–58. | Liao HF, Hwang AW, Schiariti V, Yen CF, Chi WC, Liou TH, et al. Validating the ICF core set for cerebral palsy using a national disability sample in Taiwan. Disabil Rehabil. 2020 Feb 27;42(5):642–50. |  |
| **Spinal cord injury** |  |  |  |  | Chen HC, Yen TH, Chang KH, Lin YN, Wang YH, Liou TH. Developing an ICF core set for sub-acute stages of spinal cord injury in Taiwan: A preliminary study. Disabil Rehabil. 2015 Jan 1;37(1):51–5. | Tatlı HU, Köseoğlu BF, Özcan DS, Akselim SK, Doğan A. Validation and application of the international classification of functioning core set for spinal cord injury in the Turkish patients. Turk J Phys Med Rehabil. 2019;65(3):244–58.  Herrmann KH, Kirchberger I, Stucki G, Cieza A. The comprehensive ICF core sets for spinal cord injury from the perspective of occupational therapists: A worldwide validation study using the Delphi technique. Spinal Cord. 2011;49(5):600–13. |  |
| **Traumatic brain injury** | Bernabeu M, Laxe S, Lopez R, Stucki G, Ward A, Barnes M, et al. Developing core sets for persons with traumatic brain injury based on the international classification of functioning, disability, and health. Vol. 23, Neurorehabilitation and Neural Repair. 2009. p. 464–7. |  | Aiachini B, Pisoni C, Cieza A, Cazzulani B, Giustini A, Pistarini C, et al. Developing ICF core set for subjects with traumatic brain injury: an Italian clinical perspective. Vol. 46. 2010.  Karhula ME, Kanelisto KJ, Ruutiainen J, Hämäläinen PI, Salminen AL. The activities and participation categories of the ICF Core Sets for multiple sclerosis from the patient perspective. Disabil Rehabil. 2013 Mar;35(6):492–7. |  | Laxe, S., Cieza, A., & Castaño-Monsalve, B. (2015). Rehabilitation of traumatic brain injury in the light of the ICF. NeuroRehabilitation, 36(1), 37–43. https://doi.org/10.3233/NRE-141189 | Pistarini C, Aiachini B, Coenen M, Pisoni C. Functioning and disability in traumatic brain injury: The Italian patient perspective in developing ICF Core Sets. Disabil Rehabil. 2011;33(23–24):2333–45. |  |
| **Multiple sclerosis** | Khan F, Pallant JF. Use of the International Classification of Functioning, Disability and Health (ICF) to identify preliminary comprehensive and brief core sets for multiple sclerosis. Disabil Rehabil. 2007;29(3):205–13. |  | Karhula ME, Kanelisto KJ, Ruutiainen J, Hämäläinen PI, Salminen AL. The activities and participation categories of the ICF Core Sets for multiple sclerosis from the patient perspective. Disabil Rehabil. 2013 Mar;35(6):492–7. |  | Coenen M, Cieza A, Freeman J, Khan F, Miller D, Weise A, et al. The development of ICF Core Sets for multiple sclerosis: Results of the International Consensus Conference. J Neurol. 2011 Aug;258(8):1477–88. | Conrad A, Coenen M, Schmalz H, Kesselring J, Cieza A. Validation of the Comprehensive ICF Core Set for Multiple Sclerosis From the Perspective of Physical Therapists [Internet]. 2012.  Berno S, Coenen M, Leib A, Cieza A, Kesselring J. Validation of the Comprehensive International Classification of Functioning, Disability, and Health Core Set for multiple sclerosis from the perspective of physicians. J Neurol. 2012 Aug;259(8):1713–26.  Conrad A, Coenen M, Schmalz H, Kesselring J, Cieza A. Validation of the comprehensive ICF core set for multiple sclerosis from the perspective of occupational therapists. Scand J Occup Ther. 2012 Nov;19(6):468–87.  Renom M, Conrad A, Bascuñana H, Cieza A, Galán I, Kesselring J, et al. Content validity of the Comprehensive ICF Core Set for multiple sclerosis from the perspective of speech and language therapists. Vol. 49, International Journal of Language and Communication Disorders. Blackwell Publishing Ltd; 2014. p. 672–86. |  |
| **Mental Health** | | | | | | | |
| **Schizophrenia** |  | Gorostiaga A, Balluerka N, Guilera G, Aliri J, Barrios M. Functioning in patients with schizophrenia: a systematic review of the literature using the International Classification of Functioning, Disability and Health (ICF) as a reference. Quality of Life Research. 2017 Mar 1;26(3):531–43. |  | Barrios M, Guilera G, Selb M, Gómez-Benito J. Identification of problems in the functioning of individuals with schizophrenia from the expert perspective: an Internet-based survey. Disabil Rehabil. 2017 Sep 25;39(20):2055–62. |  | Nuño L, Barrios M, Rojo E, Gómez-Benito J, Guilera G. Validation of the ICF Core Sets for schizophrenia from the perspective of psychiatrists: An international Delphi study. J Psychiatr Res. 2018 Aug 1;103:134–41.  Nuño L, Guilera G, Barrios M, Gómez-Benito J, Abdelhamid GSM. Network Analysis of the Brief ICF Core Set for Schizophrenia. Front Psychiatry. 2022 Jun 17;13.  Nuño L, Guilera G, Coenen M, Rojo E, Gómez-Benito J, Barrios M. Functioning in schizophrenia from the perspective of psychologists: A worldwide study. PLoS One. 2019 Jun 1;14(6).  Nu ̃no L., Barrios M., Rojo E., Gomez-Benito J., Guilera G. The comprehensive Icf core set for schizophrenia from the perspective of psychiatrists: A content-validity study using the Delphi technique. European Psychiatry. 2017 Apr;41(S1):s802–3. |  |
| **Bipolar disorders** | Vieta E, Cieza A, Stucki G, Chatterji S, Nieto M, Sánchez-Moreno J, et al. Developing core sets for persons with bipolar disorder based on the International Classification of Functioning, Disability and Health. Bipolar Disord. 2007 Feb;9(1–2):16–24. | Ávila CC, Cabello M, Cieza A, Vieta E, Ayuso-Mateos JL. Functioning and disability in bipolar disorders: A systematic review of literature using the ICF as a reference. Vol. 12, Bipolar Disorders. 2010. p. 473–82. | Ávila CC, Cieza A, Anaya C, Ayuso-Mateos JL. The patients’ perspective on relevant areas and problems in the bipolar spectrum disorder: Individual interviews using the International Classification of Functioning, Disability and Health as a reference tool. Vol. 91, American Journal of Physical Medicine and Rehabilitation. 2012. |  | Ayuso-Mateos JL, Ávila CC, Anaya C, Cieza A, Vieta E. Development of the international classification of functioning, disability and health core sets for bipolar disorders: Results of an international consensus process. Disabil Rehabil. 2013;35(25):2138–46. |  |  |
| **Depression** |  |  |  |  | Cieza A, Chatterji S, Andersen C, Cantista P, Herceg M, Melvin J, et al. ICF Core Sets for depression. Vol. Supplement, Journal of Rehabilitation Medicine. 2004. p. 128–34. |  |  |
| **Other Health conditions** | | | | | | | |
| **Vertigo** |  |  | Mueller M, Schuster E, Strobl R, Grill E. Identification of aspects of functioning, disability and health relevant to patients experiencing vertigo: a qualitative study using the international classification of functioning, disability and health. Health Qual Life Outcomes [Internet]. 2012;10(75). |  | Grill E, Bronstein A, Furman J, Zee DS, Müller M. International Classification of Functioning, Disability and Health (ICF) Core Set for patients with vertigo, dizziness and balance disorders. Vol. 22, Journal of Vestibular Research: Equilibrium and Orientation. 2012. p. 261–71. |  |  |
| **Hearing loss** | Danermark B, Cieza A, Gangé JP, Gimigliano F, Granberg S, Hickson L, et al. International classification of functioning, disability, and health core sets for hearing loss: A discussion paper and invitation. Int J Audiol. 2010;49(4):256–62. | Granberg, S., Dahlström, J., Möller, C., Kähäri, K., & Danermark, B. (2014). The ICF core sets for hearing loss - Researcher perspective. Part I: Systematic review of outcome measures identified in audiological research. In *International Journal of Audiology* (Vol. 53, Issue 2, pp. 65–76).  Granberg, S., Möller, K., Skagerstrand, Å., Möller, C., & Danermark, B. (2014). The ICF core sets for hearing loss: Researcher perspective, Part II: Linking outcome measures to the international classification of functioning, disability and health (ICF). In *International Journal of Audiology* (Vol. 53, Issue 2, pp. 77–87). https://doi.org/10.3109/14992027.2013.858279 | Granberg S, Pronk M, De Swanepoel W, Kramer SE, Hagsten H, Hjaldahl J, et al. The ICF core sets for hearing loss project: Functioning and disability from the patient perspective. Int J Audiol. 2014 Nov 1;53(11):777–86. | Granberg S, Swanepoel DW, Englund U, Möller C, Danermark B. The ICF core sets for hearing loss project: International expert survey on functioning and disability of adults with hearing loss using the international classification of functioning, disability, and health (ICF). Int J Audiol. 2014;53(8):497–506. | Danermark B, Granberg S, Kramer SE, Selb M, Möller C. The creation of a comprehensive and a brief core set for hearing loss using the international classification of functioning, disability and health. Am J Audiol. 2013 Dec;22(2):323–8. | Karlsson E, Mäki-Torkko E, Widén S, Gustafsson J, Manchaiah V, Mahomed-Asmail F, et al. Validation of the Brief International Classification of Functioning, Disability and Health (ICF) core set for hearing loss: an international multicentre study. Int J Audiol. 2021;60(6):412–20.  Van Leeuwen LM, Pronk M, Merkus P, Goverts ST, Terwee CB, Kramer SE. Operationalization of the Brief ICF core set for hearing loss: An ICF-Based e-intake tool in clinical otology and audiology practice. Ear Hear. 2020;1533–44. |  |
| **Vision loss** | Billiet L, Van de Velde D, Overbury O, Van Nispen RMA. International Classification of Functioning, Disability and Health core set for vision loss: A discussion paper and invitation. British Journal of Visual Impairment. 2022;40(2). | Billiet, L., Van de Velde, D., P.A. van der Aa, H., De Baets, S., & M.A. van Nispen, ruth. (2023). *The development of an International Classification of Functioning, disability, and health (ICF) Core Set: A systematic review from 2001 to 2022*. |  |  |  |  |  |
| **Deaf blindness** | Paramasivam A, Jaiswal A, Minhas R, Holzhey P, Keyes K, Lopez R, et al. The development of the International Classification of Functioning, Disability and Health Core Sets for deafblindness: A study protocol. PLoS One. 2021 Dec 1;16. | Jaiswal, A., Paramasivam, A., Budhiraja, S., Santhakumaran, P., Gravel, C., Martin, J., Ogedengbe, T. O., James, T. G., Kennedy, B., Tang, D., Tran, Y., Coloson-Osborne, H., Minhas, R., Granberg, S., & Wittich, W. (2024). The International Classification of Functioning, Disability and Health (ICF) core sets for deafblindness, part II of the systematic review: linking data to the ICF categories. *European Journal of Physical and Rehabilitation Medicine*. https://doi.org/10.23736/S1973-9087.24.07984-X | Wittich, W., Dumassais, S., Prain, M., Ogedengbe, T. O., Gravel, C., Jaiswal, A., Minhas, R., Lopez, R., & Granberg, S. (2024). Development of core sets for deafblindness using the International Classification of Functioning, Disability, and Health: the perspectives of individuals with lived experience. *European Journal of Physical and Rehabilitation Medicine*. https://doi.org/10.23736/S1973-9087.24.08500-9 | Wittich, W., Dumassais, S., Jaiswal, A., Paramasivam, A., Budhiraja, S., Lopez, R., & Granberg, S. (2024). Development of core sets for deafblindness: an international expert survey on functioning and disability of individuals living with deafblindness using the International Classification of Functioning, Disability, and Health. *European Journal of Physical and Rehabilitation Medicine*, *60*(2), 382–390. https://doi.org/10.23736/S1973-9087.24.08188-7 |  |  |  |
| **Hand Conditions** |  |  |  |  |  | Kus S, Oberhauser C, Cieza A. Validation of the brief international classification of functioning, disability, and health (ICF) core set for hand conditions. Journal of Hand Therapy. 2012;25(3):274–87. |  |
| **Inflammatory Bowel Diseases** | Peyrin-Biroulet L, Cieza A, Sandborn WJ, Kostanjsek N, Kamm MA, Hibi T, et al. Disability in inflammatory bowel diseases: Developing ICF core sets for patients with inflammatory bowel diseases based on the international classification of functioning, disability, and health. Inflamm Bowel Dis. 2010 Feb;16(1):15–22. |  |  |  |  |  |  |
| **Sleep** | Stucki A, Cieza A, Michel F, Stucki G, Bentley A, Culebras A, et al. Developing ICF Core Sets for persons with sleep disorders based on the International Classification of Functioning, Disability and Health. Sleep Med. 2008 Jan;9(2):191–8. | Gradinger F, Köhler B, Khatami R, Mathis J, Cieza A, Bassetti C. Problems in functioning from the patient perspective using the International Classification of Functioning, Disability and Health (ICF) as a reference. J Sleep Res. 2011 Mar;20(1 PART II):171–82. | Gradinger F, Glässel A, Bentley A, Stucki A. Content comparison of 115 health status measures in sleep medicine using the International Classification of Functioning, Disability and Health (ICF) as a reference. Vol. 15, Sleep Medicine Reviews. 2011. p. 33–40.  Gradinger F, Glässel A, Gugger M, Cieza A, Braun N, Khatami R, et al. Identification of problems in functioning of people with sleep disorders in a clinical setting using the International Classification of Functioning Disability and Health (ICF) Checklist. J Sleep Res. 2011 Sep;20(3):445–53. | Gradinger F, Cieza A, Stucki A, Michel F, Bentley A, Oksenberg A, Rogers A.E, DStucki G, Gartinen M. Part 1. International Classification of Functioning, Disability and Health (ICF) Core Sets for persons with sleep disorders: Results of the consensus process integrating evidence from preparatory studies. J Sleep Med. 2011 Jan;12(1):92-96. | Gradinger F, Cieza A, Stucki A, Michel F, Bentley A, Oksenberg A, et al. Part 1. International Classification of Functioning, Disability and Health (ICF) Core Sets for persons with sleep disorders: Results of the consensus process integrating evidence from preparatory studies. Sleep Med. 2011 Jan;12(1):92–6. |  |  |
| **ADHD** | Bölte S, de Schipper E, Holtmann M, Karande S, de Vries PJ, Selb M, et al. Development of ICF Core Sets to standardize assessment of functioning and impairment in ADHD: the path ahead. Eur Child Adolesc Psychiatry. 2014 Nov 28;23(12):1139–48 | de Schipper E, Lundequist A, Wilteus AL, Coghill D, de Vries PJ, Granlund M, et al. A comprehensive scoping review of ability and disability in ADHD using the International Classification of Functioning, Disability and Health-Children and Youth Version (ICF-CY). Vol. 24, European Child and Adolescent Psychiatry. Dr. Dietrich Steinkopff Verlag GmbH and Co. KG; 2015. p. 859–72. | Mahdi S, Viljoen M, Massuti R, Selb M, Almodayfer O, Karande S, et al. An international qualitative study of ability and disability in ADHD using the WHO-ICF framework. Eur Child Adolesc Psychiatry. 2017 Oct 1;26(10):1219–31.  Mahdi S, Ronzano N, Knüppel A, Dias JC, Albdah A, Chien-Ho L, et al. An international clinical study of ability and disability in ADHD using the WHO-ICF framework. Eur Child Adolesc Psychiatry. 2018 Oct 1;27(10):1305–19. | de Schipper E, Mahdi S, Coghill D, de Vries PJ, Gau SSF, Granlund M, et al. Towards an ICF core set for ADHD: a worldwide expert survey on ability and disability. Eur Child Adolesc Psychiatry. 2015 Dec 1;24(12):1509–21. | Bölte S, Mahdi S, Coghill D, Gau SSF, Granlund M, Holtmann M, et al. Standardised assessment of functioning in ADHD: consensus on the ICF Core Sets for ADHD. Eur Child Adolesc Psychiatry. 2018 Oct 1;27(10):1261–81. | Söderström S, Pettersson R, Edlund-Söderström K, Ganse G, Holmkvist E, Westin O, et al. Development of a Swedish comprehensive international classification of functioning, disability and health (ICF) core set for adult patients with attention-deficit hyperactivity disorder (ADHD). Nord J Psychiatry. 2014;68(3):161–8. |  |
| **Autism spectrum disorder** | Bölte S, De Schipper E, Robison JE, Wong VCN, Selb M, Singhal N, et al. Classification of functioning and impairment: The development of ICF core sets for autism spectrum disorder. Autism Research. 2014;7(1):167–72. | De Schipper E, Lundequist A, Coghill D, et al.Ability and disability in autism spectrum disorder: a systematic literature review employing the International Classification of Functioning, Disability and Health-Children and Youth Version. Autisl Research. 2025; 8(7): 782-794 | Mahdi S, Albertowski K, Almodayfer O, Arsenopoulou V, Carucci S, Dias JC, et al. An International Clinical Study of Ability and Disability in Autism Spectrum Disorder Using the WHO-ICF Framework. J Autism Dev Disord. 2018 Jun 1;48(6):2148–63. | de Schipper E, Mahdi S, de Vries P, Granlund M, Holtmann M, Karande S, et al. Functioning and disability in autism spectrum disorder: A worldwide survey of experts. Autism Research. 2016 Sep 1;9(9):959–69. | Bölte, S., Mahdi, S., de Vries, P. J., Granlund, M., Robison, J. E., Shulman, C., Swedo, S., Tonge, B., Wong, V., Zwaigenbaum, L., Segerer, W., & Selb, M. (2019). The Gestalt of functioning in autism spectrum disorder: Results of the international conference to develop final consensus International Classification of Functioning, Disability and Health core sets. Autism, 23(2), 449–467. https://doi.org/10.1177/1362361318755522 |  |  |
| **Amputation lower limb** | Kohler F, Cieza A, Stucki G, Geertzen J, Burger H, Dillon MP, et al. Developing Core Sets for persons following amputation based on the International Classification of Functioning, Disability and Health as a way to specify functioning. Prosthet Orthot Int. 2009;33(2):117–29. | Xu J, Kohler F, Dickson H. Systematic review of concepts measured in individuals with lower limb amputation using the International Classification of Functioning, Disability and Health as a reference. Vol. 35, Prosthetics and Orthotics International. 2011. p. 262–8. | (60) Xu J. A Core Set for people following lower limb amputation based on the International Classification of Functioning, Disability and Health [Internet]. 2019. 1–288 p. | Xu, J. (2019). A Core Set for people following lower limb amputation based on the International Classification of Functioning, Disability and Health. https://doi.org/10.26190/unsworks/22022 |  |  |  |
| **Cancer** | | | | | | | |
| **Head and neck cancer** | Tschiesner U, Cieza A, Rogers SN, Piccirillo J, Funk G, Stucki G, et al. Developing core sets for patients with head and neck cancer based on the International Classification of Functioning, Disability and Health (ICF). Vol. 264, European Archives of Oto-Rhino-Laryngology. 2007. p. 1215–22. |  | Tschiesner U, Linseisen E, Coenen M, Rogers S, Harreus U, Berghaus A, et al. Evaluating sequelae after head and neck cancer from the patient perspective with the help of the International Classification of Functioning, Disability and Health. European Archives of Oto-Rhino-Laryngology. 2009 Mar;266(3):425–36.  Tschiesner U, Sabariego C, Linseisen E, Becker S, Stier-Jarmer M, Cieza A, et al. Priorities of head and neck cancer patients: a patient survey based on the brief ICF core set for HNC. Eur Arch Otorhinolaryngol. 2013;270(12):3133–42.  Tschiesner U, Linseisen E, Baumann S, Siedek V, Stelter K, Berghaus A, et al. Assessment of Functioning in patients with head and neck cancer according to the International Classification of Functioning, Disability, and Health (ICF): A multicenter study. Laryngoscope. 2009 May;119(5):915–23. | Tschiesner U, Becker S, Cieza A. Health Professional Perspective on Disability in Head and Neck Cancer [Internet]. Vol. 136, Arch Otolaryngol Head Neck Surg. 2010. | Tschiesner U, Rogers S, Dietz A, Yueh B, Cieza A. Development of ICF core sets for head and neck cancer. Head Neck. 2010 Feb;32(2):210–20. | Becker S, Kirchberger I, Cieza A, Berghaus A, Harréus U, Reichel O, et al. Content validation of the comprehensive ICF core set for Head and Neck Cancer (HNC): The perspective of psychologists. Psychooncology. 2010 Jun;19(6):594–605.  Leib A, Cieza A, Tschiesner U. Perspective of physicians within a multidisciplinary team: Content validation of the comprehensive ICF core set for head and neck cancer. Head Neck. 2012 Jul;34(7):956–66.  Rogers SN, Forgie S, Lowe D, Precious L, Haran S, Tschiesner U. Development of the International Classification of Functioning, Disability and Health as a brief head and neck cancer patient questionnaire. Int J Oral Maxillofac Surg. 2010 Oct;39(10):975–82.  Engelbarts M, Schuster V, Kisser U, Sabariego C, Stier-Jarmer M, Coenen M, et al. The “Comprehensive ICF Core Set for Head and Neck Cancer”: a Delphi consensus survey among German speaking speech and language therapists. European Archives of Oto-Rhino-Laryngology. 2017 Jun 1;274(6):2589–99. |  |
| **Breast cancer** |  | Brockow T, Duddeck K, Geyh S, Schwarzkopf SR, Weigl M, Franke T, et al. Identifying the concepts contained in outcome measures of clinical trials on breast cancer using the International Classification of Functioning, Disability and Health as a reference. J Rehabil Med Suppl. 2004 Jul;(44):43–8. | Cooney M, Galvin R, Connolly E, Stokes E. The International Classification of Functioning (ICF) Core Set for breast cancer from the perspective of women with the condition. Disabil Rehabil. 2013 May;35(9):740–8.  Khan F, Amatya B, Demetrios M, Pallant JF. Relevance and completeness of the international classification of functioning, disability and health (ICF) comprehensive breastcancer core set: The patient perspective in an australian community cohort. J Rehabil Med. 2012;44(7):570–80. |  | Brach M, Cieza A, Stucki G, Füßl M, Cole A, Ellerin BE, et al. ICF Core Sets for breast cancer. Journal of Rehabilitation Medicine, Supplement. 2004. p. 121–7. | Glaessel A, Kirchberger I, Stucki G, Cieza A. Does the Comprehensive International Classification of Functioning, Disability and Health (ICF) Core Set for Breast Cancer capture the problems in functioning treated by physiotherapists in women with breast cancer? Physiotherapy. 2011 Mar;97(1):33–46. |  |
| **Brain tumor** | Bernabeu, M., Laxe, S., Lopez, R., Stucki, G., Ward, A., Barnes, M., Kostanjsek, N., Reed, G., Tate, R., Whyte, J., Zasler, N., & Cieza, A. (2009). Developing core sets for persons with traumatic brain injury based on the international classification of functioning, disability, and health. In Neurorehabilitation and Neural Repair (Vol. 23, Issue 5, pp. 464–467). https://doi.org/10.1177/1545968308328725 |  | Khan F, Amatya B. Use of the international Classification of Functioning, Disability and Health (ICF) to describe patient -reported disability in primary brain tumour in an Australian comunity cohort. J Rehabil Med. 2013;45(5):434–45. |  | Shen Hsiao, S.-T., Kuo, C.-Y., Liou, T.-H., Wang, T.-T., Liu, Y.-L., & Tseng, S.-H. (2022). Developing an International Classification of Functioning, Disability and Health Core Set for Pediatric Brain Tumor Survivors in Chinese Clinical Settings Core sets. https://doi.org/10.1097/NCC.0000000000001117 |  |  |
| **Cardiovascular and Respiratory Conditions** | | | | | | | |
| **Stroke** |  | Ezekiel L, Collett J, Mayo NE, Pang L, Field L, Dawes H. Factors Associated With Participation in Life Situations for Adults With Stroke: A Systematic Review. Vol. 100, Archives of Physical Medicine and Rehabilitation. W.B. Saunders; 2019. p. 945–55. | Glässel A, Coenen M, Kollerits B, Cieza A. Validation of the extended ICF core set for stroke from the patient perspective using focus groups. Disabling Rehabil. 2012;34(2):157–66.  Paanalahti M, Murphy MA, Lundgren-Nilsson Å, Sunnerhagen KS. Validation of the Comprehensive ICF Core Set for stroke by exploring the patient’s perspective on functioning in everyday life: A qualitative study. Vol. 37, International Journal of Rehabilitation Research. Lippincott Williams and Wilkins; 2014. p. 302–10. |  | Geyh S, Cieza A, Schouten J, Dickson H, Frommelt P, Omar Z, et al. ICF Core Sets for stroke. Vol. suppl, Journal of Rehabilitation Medicine. 2004. p. 135–41. | Huang, S. W., Lin, L. F., Chang, K. H., Escorpizo, R., & Liou, T. H. (2020). Development of a comprehensive core set from the international classification of functioning, disability and health for return to work among patients with stroke through Delphi-based consensus. European Journal of Physical and Rehabilitation Medicine, 56(3), 257–264. https://doi.org/10.23736/S1973-9087.20.05992-4  Kinoshita S, Abo M, Miyamura K, Okamoto T, Kakuda W, Kimura I, et al. Validation of the “Activity and participation” component of ICF core sets for stroke patients in Japanese rehabilitation wards. J Rehabil Med. 2016 Oct 1;48(9):764–8.  Lemberg I, Kirchberger I, Stucki G, Cieza A, Aboytes C, Abrams GM, et al. The ICF Core Set for stroke from the perspective of physicians: a worldwide validation study using the Delphi technique. Vol. 46. 2010.  Glässel A, Kirchberger I, Kollerits B, Amann E, Cieza A. Content Validity of the Extended ICF Core Set for Stroke: An International Delphi Survey of Physical Therapists. Phys Ther. 2011;91(8):1211–22.  Glässel A, Kirchberger I, Linseisen E, Stamm T, Cieza A, Stucki G. Content validation of the international classification of functioning, disability and health (icf) core set for stroke: the perspective of occupational therapists. Canadian Journal of Occupational Therapy. 2010 Dec;77(5):289–302.  Wang P, Li H, Guo Y, Xie Y, Ge R, Qiu Z. The feasibility and validity of the comprehensive ICF core set for stroke in Chinese clinical settings. Clin Rehabil. 2014 Feb;28(2):159–71. |  |
| **Obesity** |  |  | Raggi A, Sirtori A, Brunani A, Liuzzi A, Leonardi M. Use of the ICF to describe functioning and disability in obese patients. Disabil Rehabil. 2009;31. |  | Stucki A, Daansen P, Fuessl M, Cieza A, Huber E, Atkinson R, et al. ICF Core Sets for obesity. Journal of Rehabilitation Medicine, Supplement. 2004. p. 107–13. | Xie L, Wu Q, Hu W, Li W, Xiang G, Hao S, et al. Performance of brief ICF-sleep disorders and obesity core set in obstructive sleep apnea patients. Respir Res. 2020 Jun 22;21(1). |  |
| **Diabetes mellitus** |  |  | Kirchberger I, Coenen M, Hierl FX, Dieterle C, Seissler J, Stucki G, et al. Validation of the International classification of Functioning, disability and health (ICF) core set for diabetes mellitus from the patient perspective using focus groups. Diabetic Medicine. 2009 Jul;26(7):700–7. |  | Ruof J, Cieza A, Wolff B, Angst F, Ergeletzis D, Omar Z, et al. ICF Core Sets for diabetes mellitus. Journal of Rehabilitation Medicine, Supplement. 2004. p. 100–6. | Tsutsui H, Ojima T, Ozaki N, Kusunoki M, Ishiguro T, Oshida Y. Validation of the Comprehensive International Classification of Functioning, Disability and Health (ICF) Core Set for Diabetes Mellitus in patients with diabetic nephropathy. Clin Exp Nephrol. 2015 Apr 1;19(2):254–63.  Abdullah MF, Nor M, Zubaidah S, Ali M, Bukhary N, Bukhary I, et al. Validation of the Comprehensive ICF Core Sets for Diabetes Mellitus: A Malaysian Perspective. Annals Academy of Medicine. 2011;40(4):168–78.  Awad H, Alghadir A. Validation of the comprehensive international classification of functioning, disability and health core set for diabetes mellitus: Physical therapists’ perspectives. Am J Phys Med Rehabil. 2013 Nov;92(11):968–79. |  |
| **Obstructive pulmonary disease** |  |  |  |  | Stucki A, Stoll T, Cieza A, Weigl M, Giardini A, Wever D, et al. ICF Core Sets for obstructive pulmonary diseases. Journal of Rehabilitation Medicine, Supplement. 2004. p. 114–20. | Huang J, Reinhardt JD, Dai R, Wang P, Zhou M. Validation of the brief international classification of functioning, disability, and health core set for obstructive pulmonary disease in the Chinese context. Chron Respir Dis. 2019 May 2;16.  Lage SM, Jácome C, Oliveira A, Araújo AG, Pereira DAG, Parreira VF. Validation of the International Classification of Functioning, Disability and Health Core Set for obstructive pulmonary diseases in the perspective of adults with asthma. Disabil Rehabil. 2020 Jan 2;42(1):86–92.  Marques A, Jácome C, Gonçalves A, Silva S, Lucas C, Cruz J, et al. Validation of the Comprehensive ICF Core Set for obstructive pulmonary diseases from the patient’s perspective. International Journal of Rehabilitation Research. 2014;37(2):152–8.  Zhang Y, Meng X, Shen Y, Xie J, Yu X, Wang Q, et al. The reliability and validity of the brief icf core set in patients with chronic obstructive pulmonary disease. International Journal of COPD. 2021;16:2077–87.  Rauch A, Kirchberger I, Stucki G, Cieza A. Validation of the Comprehensive ICF Core Set for obstructive pulmonary diseases from the perspective of  physiotherapists. Physiotherapy Research International Physiother Res Int [Internet]. 2009;14(4):242–59.  Jobst A, Kirchberger I, Cieza A, Stucki G, Stucki A. Content Validity of the Comprehensive ICF Core  Set for Chronic Obstructive Pulmonary Diseases: An International Delphi Survey. Open Respir Med J. 2013;7:33–45. |  |
| **Cardio pulmonary conditions** |  |  |  |  | Boldt C, Grill E, Wildner M, Portenier L, Wilke S, Stucki G, et al. ICF Core Set for patients with cardiopulmonary conditions in the acute hospital. Vol. 27, Disability and Rehabilitation. 2005. p. 375–80. | Dinga X, Reimanna S, Timrottb K, Gutenbrunnera C, Schwarzea M, Fischer MJ. Comparison of the ICF core set for cardiopulmonary conditions in the acute hospital setting among different types of transplant patients. International Journal of Rehabilitation Research. 2010 Dec;33(4):325–31. |  |
| **Chronic ischemic heart disease** |  |  |  |  | Cieza A, Stucki A, Geyh S, Berteanu M, Quittan M, Simon A, et al. ICF Core Sets for chronic ischaemic heart disease. Journal of Rehabilitation Medicine, Supplement. Taylor and Francis A.S.; 2004. p. 94–9. |  |  |
| **Diverse situations** | | | | | | | |
| **Geriatric patients** |  | Tomandl J, Heinmüller S, Selb M, Graessel E, Freiberger E, Kühlein T, et al. Laying the foundation for a Core Set of the International Classification of Functioning, Disability and Health for community-dwelling older adults in primary care: Relevant categories of their functioning from the research perspective, a scoping review. BMJ Open. 2021 Feb 17;11(2). | Grill E, Stucki G, Boldt C, Joisten S, Swoboda W. Identification of relevant ICF categories by geriatric patients in an early post-acute rehabilitation facility. Vol. 27, Disability and Rehabilitation. 2005. p. 467–73. | Grill E, Quittan M, Huber EO, Boldt C, Stucki G. Identification of relevant ICF categories by health professionals in the acute hospital. Vol. 27, Disability and Rehabilitation. 2005. p. 437–45.  Gotthardt S, Tomandl J, Hoefle A, Kuehlein T, Book S, Graessel E, et al. Laying the foundation for an ICF core set for community dwelling older adults in primary care: an expert survey. Z Gerontol Geriatr. 2021 Jul 1;54(4):365–70. | Grill E, Hermes R, Swoboda W, Uzarewicz C, Kostanjsek N, Stucki G. ICF Core Set for geriatric patients in early post-acute rehabilitation facilities. Vol. 27, Disability and Rehabilitation. 2005. p. 411–7. | Starrost K, Geyh S, Trautwein A, Grunow J, Ceballos-Baumann A, Prosiegel M, et al. Interrater Reliability of the Extended ICF Core Set for Stroke Applied by Physical Therapists Background and Purpose. The World Health Organization’s International. Physican Therapy [Internet]. 2008;88(7):841–51.  Stier-Jarmer M, Grill E, Müller M, Strobl R, Quittan M, Stucki G. Validation of the comprehensive icf core set for patients in geriatric post-acute rehabilitation facilities. J Rehabil Med. 2011 Jan;43(2):113–22. |  |
| **Vocational rehabilitation** |  | Momsen AH, Stapelfeldt CM, Rosbjerg R, Escorpizo R, Labriola M, Bjerrum M. International Classification of Functioning, Disability and Health in Vocational Rehabilitation: A Scoping Review of the State of the Field. Vol. 29, Journal of Occupational Rehabilitation. Springer New York LLC; 2019. p. 241–73. |  |  | Finger, M. E., Escorpizo, R., Gälssel, A., Gmünder, H. P., Lückenkemper, M., Chan, C., Fritz, J., Studer, U., Ekholm, J., Kostanjsek, N., Stucki, G., & Cieza, A. (2012). ICF Core Set for vocational rehabilitation: Results of an international consensus conference. Disability and Rehabilitation, 34(5), 429–438. | Kaech Moll VM, Escorpizo R, Portmann Bergamaschi R, Finger ME. Validation of the Comprehensive ICF Core Set for Vocational Rehabilitation From the Perspective of Physical Therapists: International Delphi Survey [Internet]. 2016.  Reneman MF, Beemster TT, Edelaar MJA, Van Velzen JM, Van Bennekom C, Escorpizo R. Towards an ICF- and IMMPACT-based pain vocational rehabilitation core set in the Netherlands. J Occup Rehabil. 2013 Dec;23(4):576–84.  Aiachini B, Cremascoli S, Escorpizo R, Pistarini C. Validation of the ICF core set for vocational rehabilitation from the perspective of patients with spinal cord injury using focus groups. Disabil Rehabil. 2016 Feb 13;38(4):337–45.  Paltrinieri S, Costi S, Pellegrini M, Díaz Crescitelli ME, Vicentini M, Mancuso P, et al. Adaptation of the Core Set for Vocational Rehabilitation for Cancer Survivors: A Qualitative Consensus-Based Study. J Occup Rehabil. 2022 Dec 1;32(4):718–30. |  |
| **Burn injuries** |  |  |  |  | Lin YR, Wang JY, Chang SC, Chang KH, Chen HC, Escorpizo R, et al. Developing a delphi-based comprehensive core set from the international classification of functioning, disability, and health framework for the rehabilitation of patients with burn injuries. Int J Environ Res Public Health. 2021 Apr 2;18(8). |  |  |
