# Supplementary material for: Methodologies used in the development of the international classification of functioning, disability and health core sets: a systematic scoping review
Source: BMJ Open. 2026 Oct 5;16(10):e123955. doi: 10.1136/bmjopen-2026-123955 (PMC13646977; doi:10.1136/bmjopen-2026-123955)
Supplement: Supplementary file 3 [file bmjopen-16-10-s002.docx]

**Appendix 2: PubMed search strategy**

("International Classification of Functioning, Disability and Health"[Mesh]

OR ICF

OR "International Classification of Functioning")

AND

("Core Set*" OR "ICF Core Set*" OR "Core Sets")

AND

(development OR methodology OR validation OR implementation OR consensus OR survey OR review)

Filters:

- Humans
- English language
- Full text available
- Publication date from January 1, 2001 to July, 2024
